# JMJD1C forms condensate to facilitate a RUNX1-dependent gene expression program shared by multiple types of AML cells

**DOI:** 10.1093/procel/pwae059

**Published:** 2024-10-25

**Authors:** Qian Chen, Saisai Wang, Juqing Zhang, Min Xie, Bin Lu, Jie He, Zhuoran Zhen, Jing Li, Jiajun Zhu, Rong Li, Pilong Li, Haifeng Wang, Christopher R Vakoc, Robert G Roeder, Mo Chen

**Affiliations:** State Key Laboratory of Molecular Oncology, School of Basic Medical Sciences, Tsinghua University, Beijing 100084, China; State Key Laboratory of Molecular Oncology, School of Basic Medical Sciences, Tsinghua University, Beijing 100084, China; School of Life Sciences, Tsinghua-Peking Center for Life Sciences, Center for Synthetic and Systems Biology, Tsinghua University, Beijing 100084, China; School of Basic Medical Sciences, Tsinghua University, Beijing 100084, China; Cold Spring Harbor Laboratory, Cold Spring Harbor, NY 11724, United States; Nuclear Radiation Injury Protection and Treatment Department, Navy Medical Center of People Liberation Army (PLA), Second Military Medical University (Naval Medical University), Shanghai 200052, China; State Key Laboratory of Molecular Oncology, Tsinghua-Peking Center for Life Sciences, School of Basic Medical Sciences, Tsinghua University, Beijing 100084, China; Department of Precision Medicine, Changhai Hospital, Second Military Medical University (Naval Medical University), Shanghai 200433, China; State Key Laboratory of Molecular Oncology, Tsinghua-Peking Center for Life Sciences, School of Basic Medical Sciences, Tsinghua University, Beijing 100084, China; Nuclear Radiation Injury Protection and Treatment Department, Navy Medical Center of People Liberation Army (PLA), Second Military Medical University (Naval Medical University), Shanghai 200052, China; State Key Laboratory of Membrane Biology, Frontier Research Center for Biological Structure, School of Life Sciences, Tsinghua University-Peking University Joint Center for Life Sciences, Tsinghua University, Beijing 100084, China; School of Life Sciences, Tsinghua-Peking Center for Life Sciences, Center for Synthetic and Systems Biology, Tsinghua University, Beijing 100084, China; Cold Spring Harbor Laboratory, Cold Spring Harbor, NY 11724, United States; Laboratory of Biochemistry and Molecular Biology, The Rockefeller University, New York, NY 10065, United States; State Key Laboratory of Molecular Oncology, School of Basic Medical Sciences, Tsinghua University, Beijing 100084, China; SXMU-Tsinghua Collaborative Innovation Center for Frontier Medicine, Shanxi Medical University, Taiyuan 030607, China

**Keywords:** acute myeloid leukemia, JMJD1C, RUNX1, phase separation, enhancer-promoter interaction, enzymatic activity independent function

## Abstract

JMJD1C (Jumonji Domain Containing 1C), a member of the lysine demethylase 3 (KDM3) family, is universally required for the survival of several types of acute myeloid leukemia (AML) cells with different genetic mutations, representing a therapeutic opportunity with broad application. Yet how JMJD1C regulates the leukemic programs of various AML cells is largely unexplored. Here we show that JMJD1C interacts with the master hematopoietic transcription factor RUNX1, which thereby recruits JMJD1C to the genome to facilitate a RUNX1-driven transcriptional program that supports leukemic cell survival. The underlying mechanism hinges on the long N-terminal disordered region of JMJD1C, which harbors two inseparable abilities: condensate formation and direct interaction with RUNX1. This dual capability of JMJD1C may influence enhancer-promoter contacts crucial for the expression of key leukemic genes regulated by RUNX1. Our findings demonstrate a previously unappreciated role for the non-catalytic function of JMJD1C in transcriptional regulation, underlying a mechanism shared by different types of leukemias.

## Introduction

Dysregulation of transcriptional programs that control cell identity can lead to cancer. Acute myeloid leukemia (AML) is an extremely heterogeneous malignancy with various genetic alterations in transcription factors (TFs) and epigenetic factors, leading to abnormal self-renewal and proliferation of immature myeloid cells (Abdel-Wahab and Levine, 2013; Ferrara and Schiffer, 2013; Fong et al., 2014; Greenblatt and Nimer, 2014; Look, 1997). Current studies of therapeutic drug development typically target specific AML genetic abnormalities (Dohner et al., 2021). However, whether these seemingly different AML subtypes share a universal transcriptional program is not clear. Identification of such a program could help identify common vulnerabilities and thus promote future therapeutics against multiple AML subtypes.

Leukemic TFs control cancer cell state through interactions with genomic elements and subsequent recruitment of cofactors (Reiter et al., 2017; Roeder, 2019; Wang et al., 2021). Targeting interactions between TFs and cofactors has been proposed as a potential therapeutic strategy (Benyoucef et al., 2016; Piccolo et al., 2023; Wang et al., 2021). Earlier studies mainly focused on the functions of the enzymatic domains of chromatin factors (CFs) in development and disease, recent studies have shown that many histone-modifying enzymes have important roles beyond their catalytic activities (Mishra et al., 2014; Morgan and Shilatifard, 2023; Ohguchi et al., 2018; Shpargel et al., 2012), indicating the importance of their catalytically independent functions in regulating target gene expression. Thus, understanding how nonenzymatic functions of CFs contribute to cancer cell survival is important for designing therapeutic intervention.

JMJD1C, the largest member of the Jumonji domain-containing lysine demethylase 3 (KDM3) family, is aberrantly expressed in various AML cells and is required for the survival of multiple types of leukemia (Chen et al., 2015; Sroczynska et al., 2014). While loss of JMJD1C was found to substantially decrease leukemic stem cell (LSC) frequency and induced differentiation of MLL-AF9-, HOXA9-, and AML1-ETO-driven leukemia (Chen et al., 2015; Sroczynska et al., 2014; Zhu et al., 2016), JMJD1C loss led to only minor defects in blood homeostasis and hematopoietic stem cell (HSC) self-renewal (Zhu et al., 2016). These data demonstrate that JMJD1C is essential for leukemia cell survival but largely dispensable for HSC function, presenting JMJD1C as an attractive target for therapeutic intervention. Although a recent CRISPR/Cas9 screen showed that JMJD1C’s enzymatic activity domain (JmjC domain) is critical for MLLr leukemogenesis (Izaguirre-Carbonell et al., 2019), whether the JmjC domain is universally required for all types of AML that depend on the full-length protein for survival is still unknown.

RUNX1 (AML1) is a TF involved in the formation of many hematopoietic lineages by controlling the expression of many pivotal transcription regulators (Hoogenkamp et al., 2007; Huang et al., 2008). In AML with the t(8;21) translocation, the Runt DNA-binding domain of RUNX1 is fused to the ETO protein, producing a fusion protein, RUNX1-ETO, that was originally proposed to repress RUNX1 target gene expression to block differentiation (Amann et al., 2001; Liu et al., 2006, 2007). Recent studies demonstrate that RUNX1-ETO also functions to activate gene expression by forming a large AETFC complex and interacting with coactivators such as p300, JMJD1C, CARM1, and PRMT1 (Chen et al., 2015, 2022; Shia et al., 2012; Sun et al., 2013). Interestingly, several studies have reported a role for RUNX1 in supporting survival of many types of leukemia (Ben-Ami et al., 2013; Goyama et al., 2013; Iida et al., 2022; Mill et al., 2019; Morita et al., 2017; Wilkinson et al., 2013); and this function is not limited to the t(8;21) subtype, suggesting a potentially general RUNX1 requirement for leukemia survival, similar to that for JMJD1C.

In this study, we set out to elucidate the underlying mechanism that explains the general dependency for JMJD1C by AML cells with different genetic backgrounds. Toward this goal, we first identify RUNX1 as a key TF that recruits JMJD1C to chromatin, a mechanism shared by multiple types of leukemia. Then, we show that the nonstructured N-terminus of JMJD1C directly interacts with RUNX1 and that JMJD1C and RUNX1 co-regulate a set of genes required by AML cells. Finally, we further show that the N-terminus of JMJD1C forms liquid-like, RUNX1-containing condensates, which might mediate enhancer-promoter interactions and activate downstream gene expression. In summary, we have revealed a molecular mechanism underlying the general requirement for JMJD1C in multiple types of leukemia with completely different oncogenic mutations and also uncovered how an enzymatic activity-independent function of JMJD1C regulates the function of a key TF.

## Results

### RUNX1 interacts with JMJD1C in multiple types of leukemia

**Figure 1.**
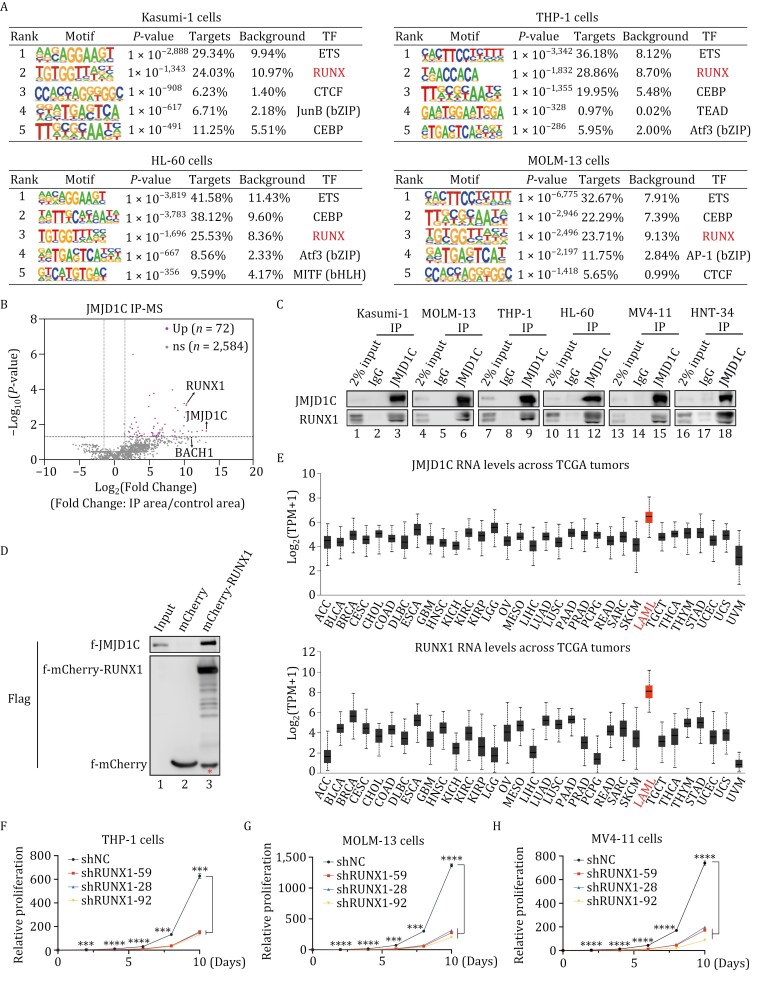
RUNX1 interacts with JMJD1C in multiple types of leukemia. (A) Motif enrichment analyses of JMJD1C ChIP-seq peaks by HOMER v4.11. *P*-values, percent enrichment of target sequences for TFs, percent of background, and best match TFs in Kasumi-1, THP-1, HL-60, and MOLM-13 cells are shown. *P*-values of motif enrichment were determined using cumulative binomial distributions by HOMER v4.11. (B) Volcano plot showing proteins co-IPed by JMJD1C compared to IgG control in four AML cell lines (HL-60, Kasumi-1, NB-4, and MOLM-13 cells). The X-axis represents the log_2_(Fold Change) (IP area/control area) and the y-axis represents −log_10_(*P*-value). (C) Immunoprecipitation of IgG or JMJD1C antibody with NEs from six AML cell lines to confirm the association of JMJD1C and RUNX1. Bound proteins were detected with antibodies shown on the left. (D) Direct interaction assay between f-JMJD1C and f-mCherrry-RUNX1 protein. Purified f-JMJD1C protein was incubated with f-mCherry or f-mCherry-RUNX1 immobilized on GST beads conjugated with GST-mCherry nanobody fusion protein. Immunoprecipitants were analyzed by immunoblot with antibodies indicated on left. *f-mCherry protein. (E) Analyses of JMJD1C (upper panel) and RUNX1 (lower panel) RNA expression levels in different types of cancer in TCGA data sets analyzed with UALCAN (Chandrashekar et al., 2017, 2022). AML, acute myeloid leukemia; ACC, adrenocortical carcinoma; BLCA, bladder urothelial carcinoma; BRCA, breast invasive carcinoma; CESC, cervical squamous cell carcinoma and endocervical adenocarcinoma; CHOL, cholangiocarcinoma; COAD, colon adenocarcinoma; DLBC, lymphoid neoplasm diffuse large B-cell lymphoma; ESCA, esophageal carcinoma; GBM, glioblastoma multiforme; HNSC, head and neck squamous cell carcinoma; KICH, kidney chromophobe; KIRC, kidney renal clear cell carcinoma; KIRP, kidney renal papillary cell carcinoma; LGG, brain lower grade glioma; OV, ovarian serous cystadenocarcinoma; MESO, mesothelioma; LIHC, liver hepatocellular carcinoma; LUAD, lung adenocarcinoma; LUSC, lung squamous cell carcinoma; PAAD, pancreatic adenocarcinoma; PRAD, prostate adenocarcinoma; PCPG, pheochromocytoma and paraganglioma; READ, rectum adenocarcinoma; SARC, sarcoma; SKCM, skin cutaneous melanoma; TGCT, testicular germ cell tumors; THCA, thyroid carcinoma; THYM, thymoma; STAD, stomach adenocarcinoma; UCEC, uterine corpus endometrial carcinoma; UCS, uterine carcinosarcoma; UVM, uveal melanoma. (F–H) Assessment of proliferation for THP-1 (F), MOLM-13 (G), and MV4-11 (H) cells treated with either control shRNA or three separate RUNX1 shRNAs. Data are presented as mean ± SD. *P*-values were determined using unpaired two-tailed Student’s *t*-test, ****P < 0.0001, ***P < 0.001.

To search for a universal transcriptional program that underlies the general dependency of JMJD1C in AML cell lines with various genetic mutations, we first performed motif enrichment analyses of JMJD1C ChIP-seq data obtained from several AML cell lines. JMJD1C-bound regions are enriched with motifs of the ETS family and RUNX family members in multiple AML cells (Figs. 1A and S1A), suggesting that JMJD1C associates with genomic regions that are directly bound by these TFs. In parallel, to identify TFs that physically associate with JMJD1C, we performed co-immunoprecipitation (co-IP) coupled with mass-spectrometry (MS). Specifically, we incubated IgG or a custom-made JMJD1C antibody (Chen et al., 2015) with nuclear extracts (NEs) derived from NB-4 (PML-RARα fusion), HL-60 (MYC overexpression), Kasumi-1 (AML1-ETO fusion), and MOLM-13 (MLL-AF9 fusion) cells. Bound proteins were resolved by sodium dodecyl sulphate–polyacrylamide gel electrophoresis (SDS–PAGE) and analyzed by MS (Figs. S1B, 1B and Table S1). Significant levels of RUNX1 were identified in the IPs in all four cell lines (Fig. 1B). We noted that BACH1 was highly enriched at three of four selected AML cells. However, BACH1 has been shown to suppress MLLr AML progression (Zhu et al., 2023b), and this function seems to be in contrast to the proposed function of JMJD1C as a dependency in AML. To reconcile this, it is possible that JMJD1C might inhibit the function of BACH1 in transcriptional repression, thereby impeding its function in impairing AML progression. Given the high ranking of the RUNX motif in JMJD1C ChIP-seq analyses and the enrichment of RUNX1 in JMJD1C IP-MS data, we focused on RUNX1 to further investigate its interaction with JMJD1C.

IPs with JMJD1C antibody in various AML cell lines confirmed JMJD1C’s association with RUNX1 (Fig. 1C). Due to the similar molecular weight of RUNX1 and IgG, we then established doxycycline (dox)-inducible mCherry-tagged RUNX1 in HL-60, MOLM-13, THP-1, and Kasumi-1 cell lines and performed IP assays with the JMJD1C antibody. The results confirmed the association of mCherry-RUNX1 and JMJD1C in all of these cell lines (Fig. S1C). Next, we set out to examine whether purified JMJD1C and RUNX1 proteins could directly interact *in vitro*. To this end, we first purified f-JMJD1C from Sf9 cells using the baculovirus system and mCherry-RUNX1 from 293T cells using the mCherry nanobody and verified their expression and purity by SDS-PAGE with Coomassie Brilliant Blue (CBB) staining (Fig. S1D). Next, *in vitro* binding experiments using mCherry nanobody and purified proteins showed that mCherry-RUNX1 directly interacts with JMJD1C (Fig. 1D).

### RUNX1 is required for the survival of JMJD1C-dependent AML cells

Next, we examined whether RUNX1 is potentially important for AML. We found that the RNA expression levels of RUNX1, like those of JMJD1C, were significantly elevated in AML samples compared to normal samples (Fig. S1E and S1F). We further analyzed the expression profiles of JMJD1C and RUNX1 across various tumor types using the TCGA database. We found that both of them are specifically highly expressed in AMLs relative to other tumor types (Fig. 1E), suggesting a potentially important role for them in AMLs. Notably, our analysis of JMJD1C and RUNX1 expression across different genetic subtypes of AML patients from the STJUDE database (McLeod et al., 2021) revealed no preferential high expression of either gene in any particular subtype within the specific mutations examined (Fig. S1G). This result supports the notion that JMJD1C and RUNX1 are essential in various types of AML cells irrespective of their mutations.

In order to examine whether RUNX1 is commonly essential for AML, we performed shRNA knockdown (KD) in several AML cell lines. Growth curves demonstrated that RUNX1, like JMJD1C (Chen et al., 2015; Sroczynska et al., 2014), is required for all the cell lines that were examined (Figs. 1F–H and S2A–C), consistent with previous findings that RUNX1 is required for AML cells with MLL fusions and AML1-ETO (Ben-Ami et al., 2013; Goyama et al., 2013; Iida et al., 2022; Mill et al., 2019; Morita et al., 2017; Wilkinson et al., 2013). Furthermore, colony-forming abilities were significantly reduced upon RUNX1 depletion in Kasumi-1, MV4-11 (MLL-AF4 fusion) and MOLM-13 cells (Fig. S2D–F). However, myeloid differentiation was not induced as assessed either by flow cytometry with the myeloid marker CD11b (Fig. S2G) or by RNA-seq analyses of differentiation markers upon RUNX KD (Fig. S2H and Table S2). To strengthen our findings, we analyzed data from The Cancer Dependency Map (DepMap). We found a preferential myeloid- and lymphoid- specific growth impediment upon depletion of RUNX1 (Fig. S2I). The requirement for JMJD1C has been demonstrated by shRNA knockdown and CRISPR/Cas9-mediated knockout analyses in various AML cell lines, including MLL-AF9, MLL-AF4, PML-RARα, and AML1-ETO, that harbor different translocations (Chen et al., 2015; Izaguirre-Carbonell et al., 2019; Sroczynska et al., 2014). However, insufficient depletion of JMJD1C might explain the negative dependency data on JMJD1C depletion in all cancer cell lines shown on Depmap. In summary, RUNX1 is essential for the survival of AML cells that depend on JMJD1C.

### JMJD1C is recruited by RUNX1 to genomic loci

**Figure 2.**
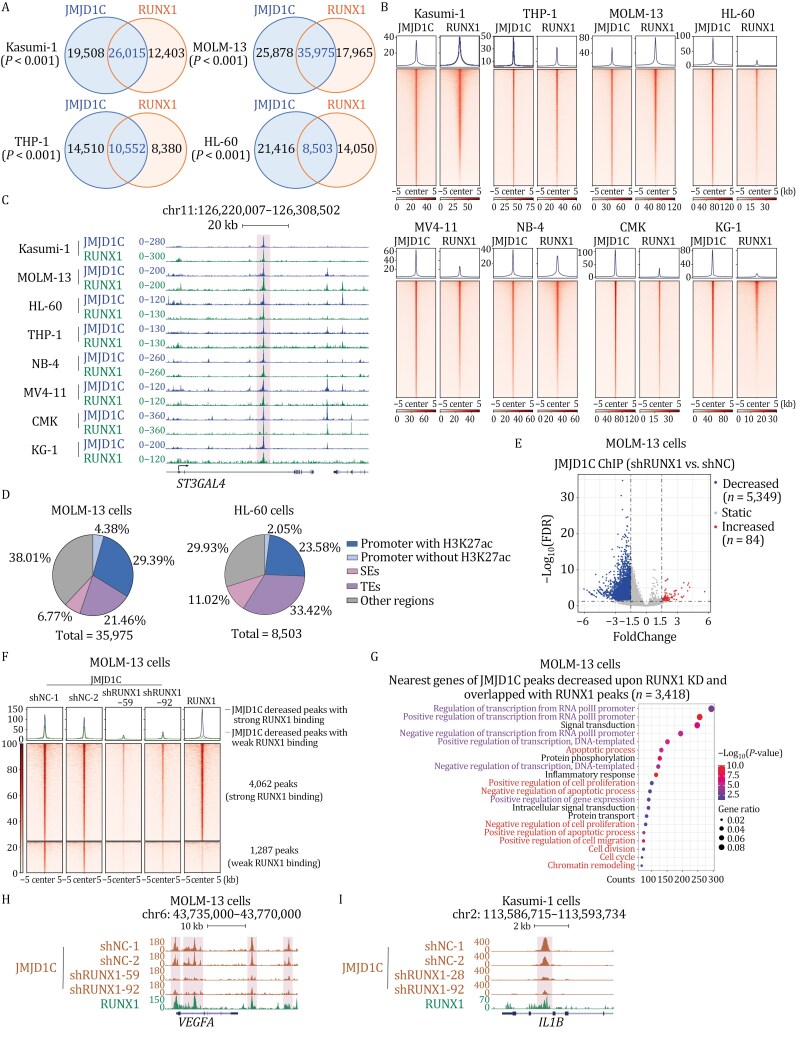
JMJD1C functions as a general cofactor for RUNX1 and is recruited by RUNX1 to genomic loci. (A) Venn diagrams showing the number of overlapped peaks from JMJD1C and RUNX1 ChIP-seq in Kasumi-1, MOLM-13, THP-1, and HL-60 cells. *P*-values were calculated by R package ChIPseeker v1.22.1. (B) Heatmaps of ChIP-seq reads for JMJD1C and RUNX1, rank-ordered from high to low by JMJD1C occupancy centered in a ±5 kb window around the JMJD1C peak center in eight AML cell lines. Color density reflects read density. (C) ChIP-seq tracks for JMJD1C and RUNX1 at the *ST3GAL4* gene locus. One intron region co-bound by JMJD1C and RUNX1 is shaded. Track names are indicated on left. The gene name is shown below the snapshot. (D) Pie charts depicting JMJD1C and RUNX1 co-bound peaks classified by different genomic regions including promoters with or without H3K27ac signal, SEs, and TEs in MOLM-13 (left panel) and HL-60 (right panel) cells. Promoters were defined as regions within 2.5 kb of transcription start sites (TSS). Peak percentages of each group are indicated. (E) Volcano plot showing the JMJD1C ChIP-seq peaks with decreased, increased, or static signals upon RUNX1 KD in MOLM-13 cells (shRUNX1 vs. shNC). (F) Heatmaps of JMJD1C and RUNX1 ChIP-seq reads for JMJD1C peaks decreased after RUNX1 depletion. Heatmaps are rank-ordered from high to low by JMJD1C occupancy in the shNC-1 group centered in a ±5 kb window around the peak center in MOLM-13 cells. Color density reflects read density. JMJD1C peaks decreased upon RUNX1 KD are further divided into two groups with either strong RUNX1 binding or weak RUNX1 binding and the two groups were separately plotted. Strong RUNX1 binding: JMJD1C peaks decreased upon RUNX1 KD that overlap with RUNX1 ChIP-seq peaks identified by MACS2 v2.1.1 with default parameters; weak RUNX1 binding: JMJD1C peaks decreased upon RUNX1 KD that does not have RUNX1 ChIP-seq peaks analyzed by MACS2 v2.1.1. (G) Gene Ontology (GO) analysis of genes with nearby JMJD1C peaks that decreased upon RUNX1 KD (shRUNX1 vs. shNC) and overlapped with RUNX1 peaks in MOLM-13 cells. (H-I) ChIP-seq tracks for JMJD1C and RUNX1 at *VEGFA* (H) and *IL1B* (I) gene loci showing JMJD1C peaks decreased upon RUNX1 KD (shRUNX1 vs. shNC) and bound by RUNX1 in MOLM-13 cells and Kasumi-1 cells. Track names are indicated on left. Gene names are shown below the snapshot.

After establishing both physical and functional interactions between JMJD1C and RUNX1, we set out to examine whether JMJD1C, which could potentially function as a RUNX1 cofactor, could be recruited to chromatin by RUNX1 and thereby co-maintain leukemic transcriptional programs. To this end, we first analyzed our own and published (Feld et al., 2018; Mandoli et al., 2016; Pencovich et al., 2011; Prange et al., 2017; Singh et al., 2017; Thoms et al., 2021) ChIP-seq data for RUNX1; and Venn diagrams show that JMJD1C ChIP peaks and RUNX1 ChIP peaks overlap significantly in all cell types examined (Figs. 2A and S3A). Furthermore, heatmaps indicate that RUNX1 binding is enriched at JMJD1C peak centers in various AML cells and vice versa (Figs. 2B and S3B). Snapshots for JMJD1C and RUNX1 co-bound genomic regions are shown for the *MYC* gene promoter and the *ST3GAL4* gene intron (Figs. 2C and S3C).

We next set out to examine where JMJD1C and RUNX1 co-colocalize in the genome. Annotation of their co-bound regions shows that they bind both promoters and other potential cis-regulatory elements (Fig. S3D). Since super-enhancers (SEs) have been shown to drive high levels of oncogenic gene expression to maintain cancer cell survival (Hnisz et al., 2013; Loven et al., 2013), we next set out to determine whether JMJD1C and RUNX1 regulate gene expression by binding to SEs. We first defined SEs (Fig. S3E–G) and typical enhancers (TEs) with H3K27ac ChIP-seq data (Chen et al., 2022; Ochi et al., 2020; Wan et al., 2017) and then identified JMJD1C and RUNX1 co-bound SEs and TEs. Pie charts show that more than half of JMJD1C and RUNX1 co-bound regions are located on active cis-regulatory elements that include SEs, TEs, and promoters with H3K27ac signals in MOLM-13, HL-60, and Kasumi-1 cells (Figs. 2D and S3H).

Next, we set out to examine if RUNX1 can recruit JMJD1C to chromatin via direct physical interaction by performing JMJD1C ChIP-seq in AML cells with RUNX1 depletion. Volcano plots show that ChIP signals of thousands of JMJD1C peaks are decreased when RUNX1 is absent while only about 100 peaks are increased (Figs. 2E and S4A), indicating that RUNX1 is essential for JMJD1C recruitment and JMJD1C may function as a cofactor for RUNX1. In addition, we found that almost all of the peaks with reduced JMJD1C binding showed RUNX1 ChIP-seq peaks, suggesting this is a direct effect of RUNX1 depletion (Figs. 2F and S4B). Notably, there were more decreased JMJD1C peaks after RUNX1 KD in MOLM-13 cells compared to Kasumi-1 cells (5,349 vs. 1,391). This could be explained by the presence of AML1-ETO in Kasumi-1 cells as our previous study showed that JMJD1C could also be recruited by AML1-ETO (Chen et al., 2015). Next, we performed GO analyses of genes near JMJD1C peaks that are decreased upon RUNX1 depletion and found that these genes are enriched for transcriptional regulation by RNA polymerase II and for biological processes related to leukemic state maintenance, such as cell proliferation, cell cycle, cell division, and apoptosis (Figs. 2G and S4C). Snapshots for several leukemia-associated genes are shown (Figs. 2H, 2I and S4D–F). In conjunction with the demonstration of direct interactions (Fig. 1D), these data indicate that RUNX1 can recruit JMJD1C to the genome through direct physical interaction and that JMJD1C functions as a cofactor of RUNX1 to control genes important for leukemic program maintenance.

### JMJD1C interacts with RUNX1 through its N-terminus

**Figure 3.**
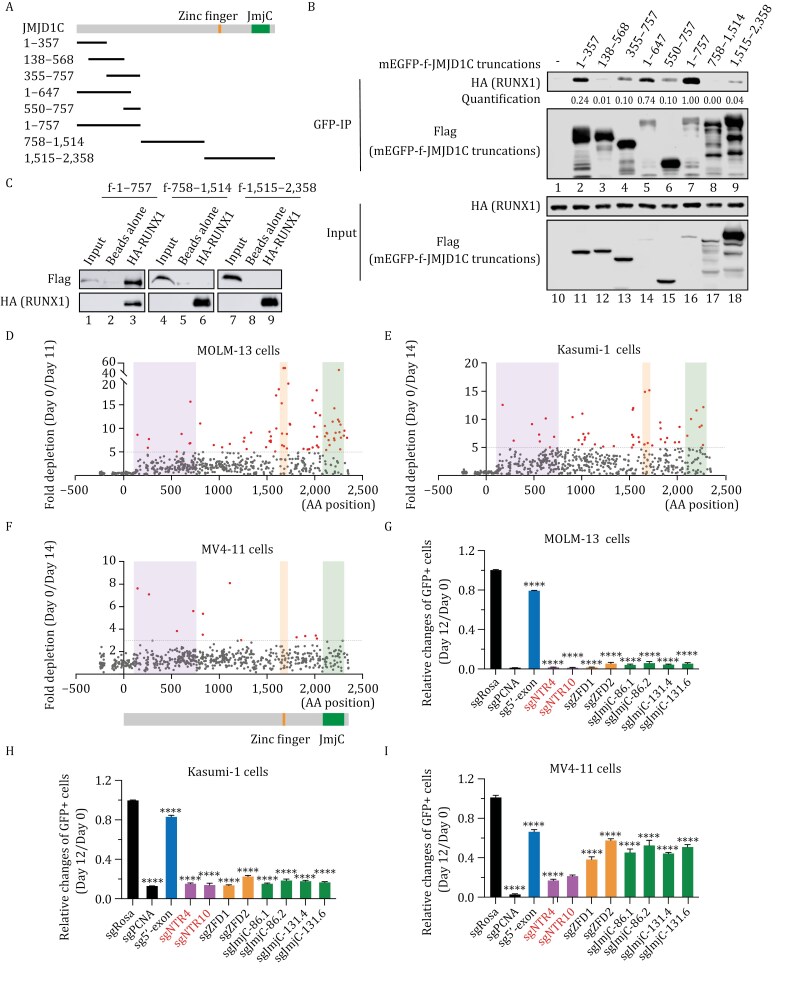
N-terminal region of JMJD1C interacts with RUNX1 and is critical for survival of multiple AML cells. (A) Schematics of different truncations of JMJD1C protein used in Fig. 3B. (B) Immunoprecipitation of GFP nanobody-conjugated beads with 293T NEs expressing different mEGFP-f-JMJD1C truncations (described in Fig. 3A) and HA-RUNX1. Bound proteins were detected with antibodies shown on left. Quantified and normalized values of co-IPed HA-RUNX1 are indicated below each lane. The co-IPed HA-RUNX1 value was determined by the HA signals in the IP group normalized by HA-RUNX1 in the input group, then further normalized to the bait mEGFP-f-JMJD1C signals. The value in Lane 7 was set as “1”. (C) Direct interaction assay using purified f-JMJD1C truncations and HA-RUNX1 protein. Purified f-JMJD1C truncated proteins were incubated with HA-RUNX1 immobilized on anti-HA agarose beads. Immunoprecipitants were analyzed by immunoblot with antibodies indicated on left. (D–F) Fold changes in GFP percentages (Day 0/Day 11 or Day 0/Day 14) of MOLM-13 (D), Kasumi-1 (E), and MV4-11 (F) cells. Day 0: the day when sgRNA expressing cells reach maximal levels of GFP expression, and when Cas9 was induced with doxycycline. Each dot represents an individual sgRNA targeting *JMJD1C* gene. Shaded areas are regions with depleted sgRNAs targeting N-terminal region of JMJD1C, zinc finger domain, and JmjC domain of JMJD1C. (G–I) Bar plots of growth competition assays showing the changes of GFP^+^ cells expressing sgRNAs targeting *Rosa*, *PCNA*, 5ʹ-exon, N-terminal region (NTR), Zinc finger domain (ZFD), and JmjC domain of JMJD1C in MOLM-13 (G), Kasumi-1 (H) and MV4-11 (I) cells. sgRNA targeting *Rosa* serves as a negative control and sgRNA targeting *PCNA* serves as a positive control. Y-axis represents the GFP percentages of Day 12 divided by Day 0 after sgRNA-expressing cells reach maximal levels of GFP expression, at which point Cas9 was induced with doxycycline. Data are presented as mean ± SD. *P*-values were determined using unpaired two-tailed Student’s *t*-test, ****P < 0.0001.

Since RUNX1 directly interacts with JMJD1C and recruits it to chromatin, we next set out to examine which part of JMJD1C interacts with RUNX1. To this end, we generated three JMJD1C truncations of approximately equal length: 1–757, 758–1,514, and 1,515–2,358 (Fig. 3A). We then transfected 293T cells with vectors expressing both HA-RUNX1 and individual monomeric enhanced GFP (mEGFP)-tagged JMJD1C truncations and performed co-IP assays using GFP nanobody. JMJD1C (1–757) associates most strongly with RUNX1 compared to the other two JMJD1C truncation mutants, indicating that the JMJD1C N-terminus is crucial for RUNX1 interaction (Fig. 3B, lanes 7–9 and 16–18). Next, we further divided the 1–757 truncation to generate five different truncations (Fig. 3A), fused them to mEGFP, and then performed similar co-IP experiments with GFP nanobody. Notably, none of the smaller truncation mutants, except for 1–647, were associated with RUNX1 almost as strongly as JMJD1C (1–757) (Fig. 3B, lanes 2–6 and 11–15), indicating that almost the entire N-terminus (the 1–757 part of JMJD1C) is required to associate with RUNX1. In order to demonstrate that the 1–757 region of JMJD1C directly interacts with RUNX1, we performed *in vitro* binding assays with purified f-JMJD1C truncation mutants and HA-RUNX1. HA-RUNX1 was purified from 293T cells, immobilized on anti-HA agarose beads, and incubated with f-JMJD1C (1–757) purified from Sf9 insect cells. As expected, f-JMJD1C (1–757) was found to directly interact with RUNX1 (Figs. 3C and S5A).

### The N-terminal region of JMJD1C is critical for AML cell survival

Next, we asked whether disruption of the JMJD1C N-terminal region that interacts with RUNX1 could lead to cell death of various AML cells. It has been reported that the JMJD1C C-terminus contains a catalytic Jumonji (JmjC) domain (Klose et al., 2006) and a zinc finger domain (ZFD) that is implicated in determining substrate specificity (Brauchle et al., 2013; Yamane et al., 2006). Considering that the JmjC domain was demonstrated to be critical for the t(8;21) AML survival by functioning as a cofactor for AML1-ETO (Chen et al., 2015), we first examined if the JmjC domain is also critical for other types of leukemia. Pooled screening of JmjC domain-targeting sgRNAs in different AML cells indicated that, of those tested, only MOLM-13 and NOMO-1 are sensitive to JmjC domain disruption, while HEL, U-937, THP-1, and SET-2 are insensitive to JmjC domain targeting (Fig. S5B; Table S3). However, most of these cells have been demonstrated to be sensitive to JMJD1C depletion by shRNAs (Chen et al., 2015; Sroczynska et al., 2014), suggesting that the non-enzymatic function of JMJD1C must be responsible for the shared requirement of these AML cells for JMJD1C.

JMJD1C is close to 300-kDa in size, with the only identified domains being the small JmjC and zinc finger domains at the C-terminus and the vast majority of the protein having unidentified functions. Since the JmjC domain is non-essential for the survival of all AML types that are dependent on full-length JMJD1C (shRNA knockdown experiments), we next performed CRISPR-Cas9 domain screening (Shi et al., 2015) to identify functionally important domains within JMJD1C that are commonly required for AML survival. We designed 610 tiling sgRNAs to cover the coding region of the *JMJD1C* gene, cloned the sgRNAs into a lentiviral vector with a GFP marker gene, and transduced pooled lentivirus into dox-inducible Cas9-expressing AML cells. After dox treatment, different positions of the *JMJD1C* gene targeted by sgRNAs will generate mutations. Functionally important domains are much less tolerant of in-frame mutations than are noncritical domains, resulting in the depletion of cells bearing the single sgRNAs targeting critical domains (Fig. S5C). Our screening experiments showed that Kasumi-1 and MOLM-13 cells are much more sensitive to sgRNAs targeting JmjC and zinc finger domains than to other sgRNAs, while MV4-11 cells are not (Fig. 3D–F, shaded with light orange and green, Table S4). Interestingly, these three AML cell lines are all responsive to sgRNAs targeting the N-terminal region of JMJD1C (Fig. 3D–F, shaded in light pink, Table S4).

To validate these results, we cloned several sgRNAs targeting the JMJD1C N-terminal region (those with more than three-fold depletion in our CRIPSR screening data), the zinc finger, and JmjC domains of JMJD1C, the 5ʹ exon of *JMJD1C*, *Rosa* (negative control), and *PCNA* (positive control) and then performed growth competition assays. Consistent with our screening results, and despite differences in their sensitivities toward JmjC and zinc finger domains-targeting sgRNAs, all cell lines tested were sensitive to sgRNAs targeting the JMJD1C N-terminal domain (Figs. 3G–I and S5D–H), further indicating the functional role of the JMJD1C N-terminal region in maintaining AML cell survival. In summary, the above findings demonstrate that the JMJD1C N-terminus directly interacts with RUNX1 and that its disruption impairs survival and growth of various AML cells. Our findings suggest that the physical interaction between RUNX1 and the JMJD1C N-terminus may explain the functional importance of JMJD1C in leukemia.

### The N-terminal region of JMJD1C forms condensates

**Figure 4.**
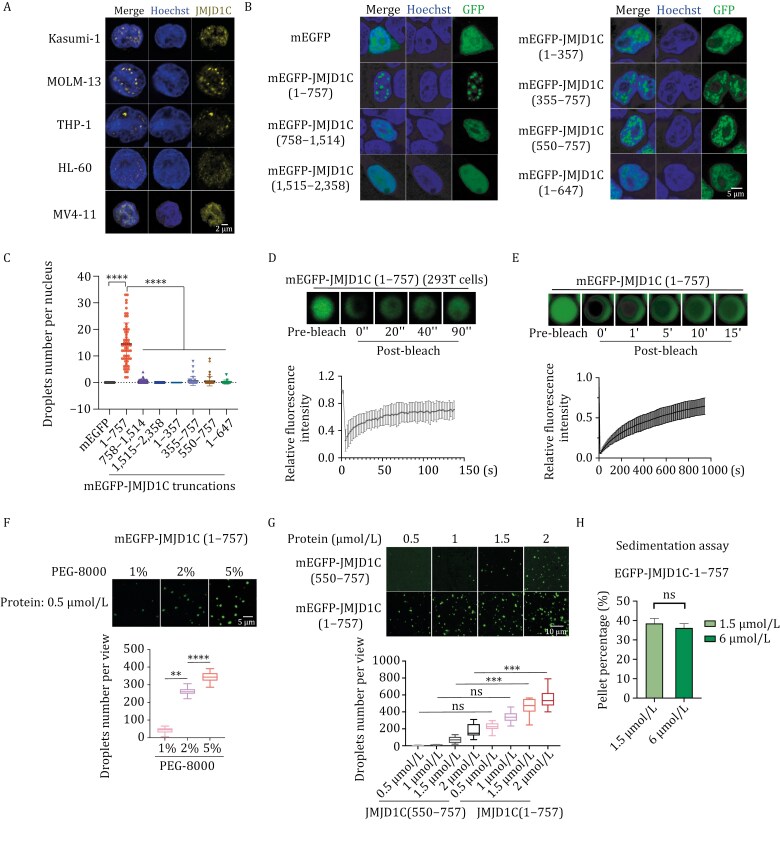
The N-terminal region of JMJD1C forms condensates. (A) Representative images for immunofluorescence staining of JMJD1C in Kasumi-1, MOLM-13, THP-1, HL-60, and MV4-11 cells. The scale bar represents 2 μm. (B) Representative images of 293T cells transfected with indicated mEGFP-tagged JMJD1C truncations. The scale bar represents 5 μm. (C) Quantification of droplet numbers per nucleus of 293T cells transfected with indicated mEGFP-tagged JMJD1C truncations as shown in Fig. 4B. *P*-values were determined using ANOVA followed by Dunn’s multiple comparison test, ****P < 0.0001. (D) Representative images of live-cell imaging of 293T cells transfected with mEGFP-JMJD1C (1–757) after photobleaching (upper panel) and quantification (lower panel). Y-axis represents relative fluorescence intensity, with a pre-bleaching signal set as “1”. X-axis, time after photobleaching. Data are shown as average relative intensity ± SD (*n* = 7). (E) Representative images of mEGFP-JMJD1C (1–757) *in vitro* FRAP assay (upper panel) and the quantification (lower panel). Y-axis represents relative fluorescence intensity, with pre-bleaching signal set as “1”. X-axis, time after photobleaching. Data are shown as average relative intensity ± SD (*n* = 9). (F) Representative images of mEGFP-JMJD1C (1–757) in droplet formation assay at different PEG-8000 concentrations (upper panel) and the quantification (lower panel). Y-axis represents droplets number per view at indicated PEG-8000 concentrations. The scale bar represents 5 μm. *P*-values were determined using Kruskal–Wallis One-Way ANOVA test, ****P < 0.0001, **P < 0.01. (G) Representative images of mEGFP-JMJD1C (550–757) and mEGFP-JMJD1C (1–757) in droplet formation assay at different protein concentrations (upper panel) and the quantification (lower panel). The y-axis represents droplets number per view at indicated protein concentrations. 2% PEG-8000 was used in the reaction. The scale bar represents 10 μm. *P*-values were determined using Kruskal–Wallis One-Way ANOVA test, ***P < 0.001, ns represents not significant. (H) Quantification of the percentages of EGFP-JMJD1C (1–757) in the condensed phase fractions in the sedimentation assay, as shown in Fig. S6G. Data are presented as mean ± SD. *P*-values were determined using unpaired two-tailed Student’s *t*-test, ns represents not significant.

Intrinsically disordered regions (IDRs) are often involved in transient protein–protein interactions and condensate formation (Ahn et al., 2021; Sabari et al., 2018; Shin and Brangwynne, 2017; Zhu et al., 2023a). CRISPR-Cas9 domain screening is more likely to identify domains with well-structured domains than nonstructured regions (Shi et al., 2015). We thus reason that this is possibly why the number of the N-terminus-targeting sgRNA hits in our domain screening were relatively small and the “fold depletion” was less significant than the JmjC domain-targeting sgRNAs. The relatively less contracted sgRNA hits could be thereby ascribed to the biophysical property of the N-terminal region of JMJD1C. Thus, we analyzed JMJD1C amino acid sequences using predictor of natural disordered regions (PONDR; Xue et al., 2010) and found that the JMJD1C N-terminal region contains a large IDR (Fig. S6A), suggesting a potential condensate formation ability. To investigate whether JMJD1C displays liquid–liquid phase separation (LLPS), we performed an immunofluorescence (IF) assay to detect endogenous JMJD1C protein in AML cells. JMJD1C was observed in nuclear condensate-like structures in Kasumi-1, MOLM-13, THP-1, HL-60, and MV4-11 cells (Figs. 4A, S6B and S6C). To determine which domains in JMJD1C are required for formation of condensates, we expressed three JMJD1C truncations (1–757, 758–1,514, and 1,515–2,358) tagged with mEGFP in 293T cells. JMJD1C (1–757), but not the other two truncations, was able to form nuclear puncta (Figs. 4B, 4C and S6D).

Phase-separated condensates are fluid-like structures with highly dynamic behaviors (Alberti et al., 2019; Jiang et al., 2020), so next we sought to determine whether these puncta are indeed liquid-like phase-separated condensates. First, we observed fusion events of mEGFP-JMJD1C (1–757) droplets in 293T cells (Fig. S6E). Next, we quantified fluorescence signals of mEGFP-JMJD1C (1–757) after photobleaching (Bolognesi et al., 2016), and observed recovery of signals within 20s (Fig. 4D). These data indicated that puncta-containing mEGFP-JMJD1C (1–757) protein has the high fluidity of nuclear condensates as fluorescence signals in protein aggregates recover only over a period of time span after photobleaching. Next, we purified mEGFP-JMJD1C (1–757) following baculovirus-mediated expression in Sf9 cells, performed FRAP assay, and found that fluorescence signals from *in vitro* formed mEGFP-JMJD1C (1–757) droplets also recovered within 60 s after photobleaching (Figs. 4E and S6F). Furthermore, we found that pure mEGFP-JMJD1C (1–757) could form droplets at a low concentration (0.5 μmol/L) *in vitro* and that the droplet numbers increase as the concentration of PEG-8000 increases (Fig. 4F and 4G). Next, we utilized a sedimentation assay to separate the condensed liquid phase from the bulk aqueous solutions via centrifugation. We then assessed the protein components in each fraction using SDS–PAGE with CBB staining and revealed that approximately 40% of JMJD1C (1–757) proteins at both high (6 µmol/L) and low concentrations (1.5 µmol/L) are present in the condensed liquid phase (Figs. 4H and S6G), further confirming JMJD1C’s ability to form condensates.

Further analyses of JMJD1C (1–757) and derived 1–357, 355–757, and 550–757 fragments indicated that only the C-terminal regions of JMJD1C (1–757), JMJD1C (355–757), and JMJD1C (550–757), retained some ability to form nuclear condensates, while the RUNX1-interacting truncation, JMJD1C (1–647), completely lost LLPS ability (Figs. 4B, 4C and S6D). Next, we chose the smaller truncation JMJD1C (550–757) to examine its *in vitro* droplet formation ability. Purified mEGFP-JMJD1C (550–757) could form droplets and the droplets could fuse *in vitro* (Fig. S6F and S6H), indicating the high fluidity of condensates. Similarly, fluorescence signals of purified mEGFP-JMJD1C (550–757) recovered within 50 s after photobleaching (Fig. S6I). However, direct comparisons showed that mEGFP-JMJD1C (1–757) formed more droplets compared to JMJD1C (550–757) under the same protein concentrations (Fig. 4G). The above results indicate that JMJD1C has the ability to form droplets both *in vivo* and *in vitro*, with its N-terminus (1–757) being crucial for condensate formation.

### The N-terminal IDR of JMJD1C is functionally promiscuous

Since RUNX1 interaction and puncta forming abilities are both located in the 1–757 region of JMJD1C, we wanted to separate the two functions into two distinct physical regions by deleting increasing numbers of amino acids from both the N- and C-termini of JMJD1C (1–757) (Fig. S7A). Since JMJD1C (1–647) maintains most of RUNX1-interacting ability while JMJD1C (550-757) maintains some of the droplet formation ability, we wanted to see if we could identify a relatively small region in the N-terminus of JMJD1C (1–757) that interacts with RUNX1. However, further truncation of JMJD1C (1–757) dramatically reduced RUNX1 binding, with JMJD1C (1–137) maintaining some ability to interact with RUNX1 (Fig. S7B, lane 8). Therefore, we next sequentially deleted 10-20 amino acids blocks from the N-terminus of JMJD1C (1–757) (Fig. S7A), hoping to obtain a truncation that maintains the droplet formation ability, which is skewed toward the C-terminus of JMJD1C (1–757) but loses the RUNX1 interaction ability. However, droplet formation and co-IP assays in 293T cells indicated the futility of our endeavor to completely separate RUNX1 binding and LLPS regions, as deletion of 30 amino acids from the N-terminus of JMJD1C (1–757) simultaneously disrupted droplet formation and RUNX1 interaction (Fig. S7C–E). Therefore, these results suggest that while the C-terminus of JMJD1C (1–757) is more important for LLPS and the N-terminus is more important for RUNX1 interaction, the full-length JMJD1C (1–757) is required for its full potential for both RUNX1 interaction and LLPS. Therefore, we believe that the JMJD1C (1–757) IDR region is functionally promiscuous and that the entire IDR is critical for its ability to efficiently form droplets and at the same time interact with RUNX1.

Next, we wanted to examine if the LLPS ability depends on the self-interacting ability of the full-length JMJD1C (1–757). To this end, we transiently transfected 293T cells with mEGFP-f-tagged JMJD1C (1–757) and Halo-HA-tagged JMJD1C truncations and then performed IP experiments with anti-HA agarose beads. We found that mEGFP-f-JMJD1C (1–757) was associated strongly with Halo-HA-JMJD1C (1–757) as expected (Fig. S7F, lane 7). Small deletions of the Halo-HA-JMJD1C (355–757) and JMJD1C (550–757) proteins completely abolished their interactions with mEGFP-f-JMJD1C (1–757) (Fig. S7F, lane 9-10). Interestingly, the droplet formation incompetent Halo-HA-JMJD1C (1–647) maintains some, but not full, interaction with mEGFP-f-JMJD1C (1–757) (Fig. S7F, lane 8). These results confirmed that the entirety of JMJD1C (1–757) is required for its self-interaction to form droplet condensates.

### The JMJD1C N-terminus mediates the formation of RUNX1-DNA-containing droplets

**Figure 5.**
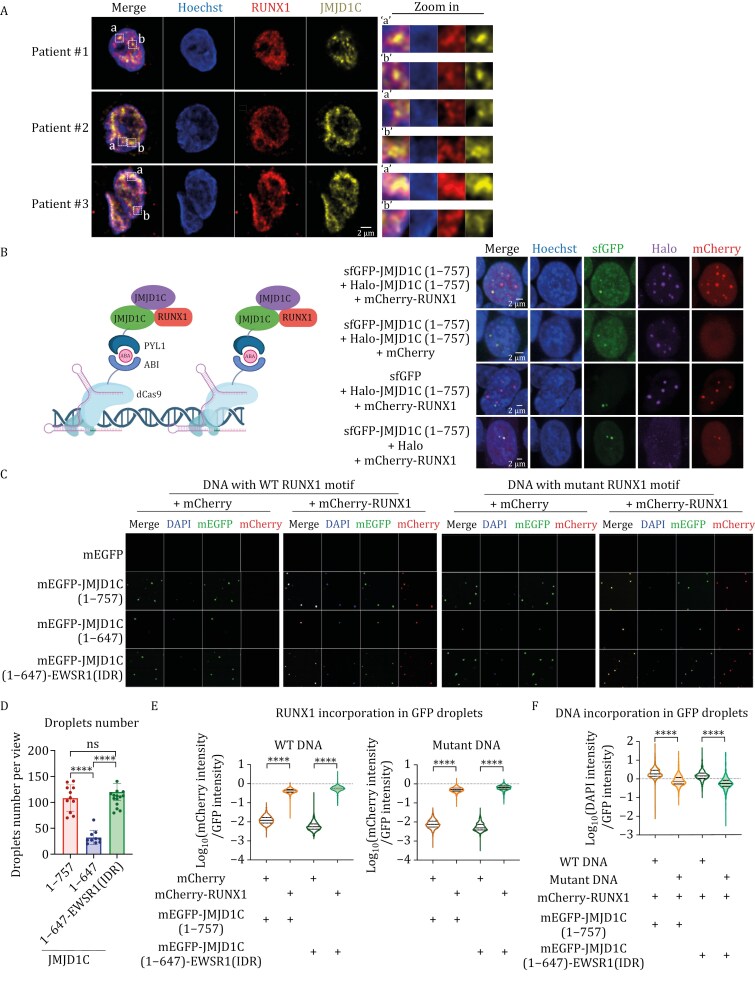
JMJD1C N-terminus mediates formation of RUNX1-DNA droplets. (A) Representative images for immunofluorescence staining of JMJD1C and RUNX1 in primary cells from three AML patients. The scale bar represents 2 μm. (B) Schematic of the ABA-inducible CRISPR-mediated genomic imaging system to visualize the self-interaction ability of JMJD1C (1–757) and its ability to recruit RUNX1 at target genomic loci (left panel, created in BioRender. C, Q. (2023)). Representative images depicting the interaction dynamics between sfGFP-JMJD1C (1–757), Halo-JMJD1C (1–757), and mCherry-RUNX1 at selected genomic loci on chromosome 3 in living 293T cells (right panel). sfGFP was fused with PYL1 and can be recruited to chromosome 3 through the dCas9-ABI system. sfGFP, Halo, or mCherry proteins serve as negative controls for sfGFP-JMJD1C (1–757), Halo-JMJD1C (1–757), or mCherry-RUNX1. The scale bars represent 2 μm. (C) Representative images of *in vitro* droplets formation assay to indicate JMJD1C N-terminus formed droplets could incorporate RUNX1 and RUNX1-bound DNA. mEGFP or mCherry proteins serve as negative controls for mEGFP-tagged proteins or mCherry-RUNX1. DNA with mutant RUNX1 motif serves as a control for non-RUNX1 binding sequence. (D) Quantification of droplet numbers formed by indicated proteins, related to Fig. 5C. *P*-values were determined using ANOVA followed by Dunn’s multiple comparison test, ****P < 0.0001, ns represents not significant. (E) Quantification of mCherry signals that are incorporated into GFP droplets. mCherry intensity was divided by GFP signal intensity in each droplet formed by mEGFP-tagged proteins to indicate the incorporation levels of mCherry-RUNX1 into condensates, related to Fig. 5C. *P*-values were determined using ANOVA followed by Dunn’s multiple comparison test, ****P < 0.0001. (F) Quantification of DAPI signals that are incorporated into GFP droplets. DAPI intensity was divided by GFP signal intensity in each mEGFP-tagged protein-formed droplet to show the incorporation levels of DNA into condensates, related to Fig. 5C. *P*-values were determined using ANOVA followed by Dunn’s multiple comparison test, ****P < 0.0001.

To explore whether JMJD1C-formed condensates could incorporate RUNX1 protein in AML cells, we performed co-IF staining for JMJD1C and RUNX1 in both AML cell lines and primary cells from three AML patients. We observed extensive co-staining of JMJD1C and RUNX, suggesting that they can form condensates *in vivo* (Figs. 5A and S8A–C). Next, we wanted to determine whether the dual properties of the JMJD1C N-terminus, namely, LLPS and RUNX1 interaction, could mediate the formation of condensates containing transcription factors, cofactors, and DNA. To this end, we utilized a CRISPR-mediated genomic imaging system, in combination with an abscisic acid (ABA)-inducible ABI/PYL1 system (Ma et al., 2016; Wang et al., 2018) (Fig. 5B, model on the left). In this system, upon ABA addition, PYL1-Superfolder GFP (sfGFP)-tagged proteins localize to the two highly repetitive (> 100×) endogenous regions within chromosome 3q29 that are targeted by sgRNA-dCas9-ABI. Then, we assayed whether Halo-tagged JMJD1C (1–757) and mCherry-tagged RUNX1 (with an R174Q point mutation disrupting its DNA binding ability) (Zhao et al., 2012) can co-localize to the same two GFP loci. Specifically, we first titrated down the transfected sfGFP-JMJD1C (1–757) plasmid so that only two GFP loci can be visualized in a single cell. Under these conditions, Halo-JMJD1C (1–757) and mCherry-RUNX1(R174Q) were recruited only to genomic loci enriched for sfGFP-JMJD1C (1–757) and not to sfGFP loci (Fig. 5B, compare the first and third rows), confirming that JMJD1C (1–757) can self-interact and recruit RUNX1 within a cell. In addition, mCherry-RUNX1(R174Q), but not mCherry, could be recruited to the sfGFP-JMJD1C (1–757) loci (Fig. 5B, compare the first and second rows). Notably, Halo-JMJD1C (1–757) could also form droplets containing mCherry-RUNX1(R174Q). These results are consistent with our earlier co-IP results (Fig. S7F) and direct protein interaction data (Fig. 1D).

Additionally, we conducted an *in vitro* binding assay by incubating purified mCherry-RUNX1 (Fig. S8D), mEGFP-JMJD1C (1–757) (Fig. S6F), and DNA with RUNX1 binding motifs. We then examined whether JMJD1C-formed droplets could incorporate RUNX1 protein and DNA. First, as a control, we found that mCherry-RUNX1 could not efficiently form condensates with mEGFP, whereas mCherry-RUNX1 and DNA could be recruited to the droplets when incubated with mEGFP-JMJD1C (1–757) (Fig. 5C–E). These results suggest that JMJD1C (1–757) is the driving force for droplet formation, bringing far away RUNX1-bound DNA into the same space. Notably, when DNA with mutated RUNX1 binding motifs was used in the assay, mEGFP-JMJD1C (1–757) still formed droplets with mCherry-RUNX1, but with no incorporation of DNA (Fig. 5C–F). These results indicate that the incorporation of DNA molecules into JMJD1C droplets requires site-specific RUNX1 binding.

Next, we examined the ability of mEGFP-JMJD1C (1–647), mCherry-RUNX1, and DNA to form droplets. JMJD1C (1–647) did not form droplets in 293T cells but could interact with RUNX1 and maintain some level of self-interaction (Figs. 3B, 4C and S7F). As expected, co-incubation of mEGFP-JMJD1C (1–647) (Fig. S8D), mCherry-RUNX1 (Fig. S8D) and DNA did not result in efficient droplet formation, confirming that the droplet formation driver is the droplet forming ability of JMJD1C (1–757) (Fig. 5C and 5D). To rescue the LLPS ability of JMJD1C (1–647), we fused the IDR of the known LLPS protein EWSR1 to mEGFP-JMJD1C (1–647) and then tested whether the resulting mEGFP-JMJD1C (1–647)-EWSR1(IDR) could recruit mCherry-RUNX1 and DNA to its droplets similarly to mEGFP-JMJD1C (1–757). To this end, we first examined the LLPS ability of mEGFP-JMJD1C (1–647)-EWSR1(IDR) and found its LLPS ability to be similar to that of mEGFP-JMJD1C (1–757) both *in vivo* and *in vitro* (Figs. 5C, 5D and S8D–F). Next, we incubated mEGFP-JMJD1C (1–647)-EWSR1(IDR) with mCherry-RUNX1 and DNA, and found that it, similarly to mEGFP-JMJD1C (1–757), could incorporate the RUNX1-DNA complex into droplets (Fig. 5C–F). The above findings indicate that the JMJD1C N-terminus mediates the formation of RUNX1-DNA droplets through its LLPS and RUNX1 interaction capabilities.

### JMJD1C and RUNX1 co-regulate leukemic transcriptional programs in multiple types of leukemia

**Figure 6.**
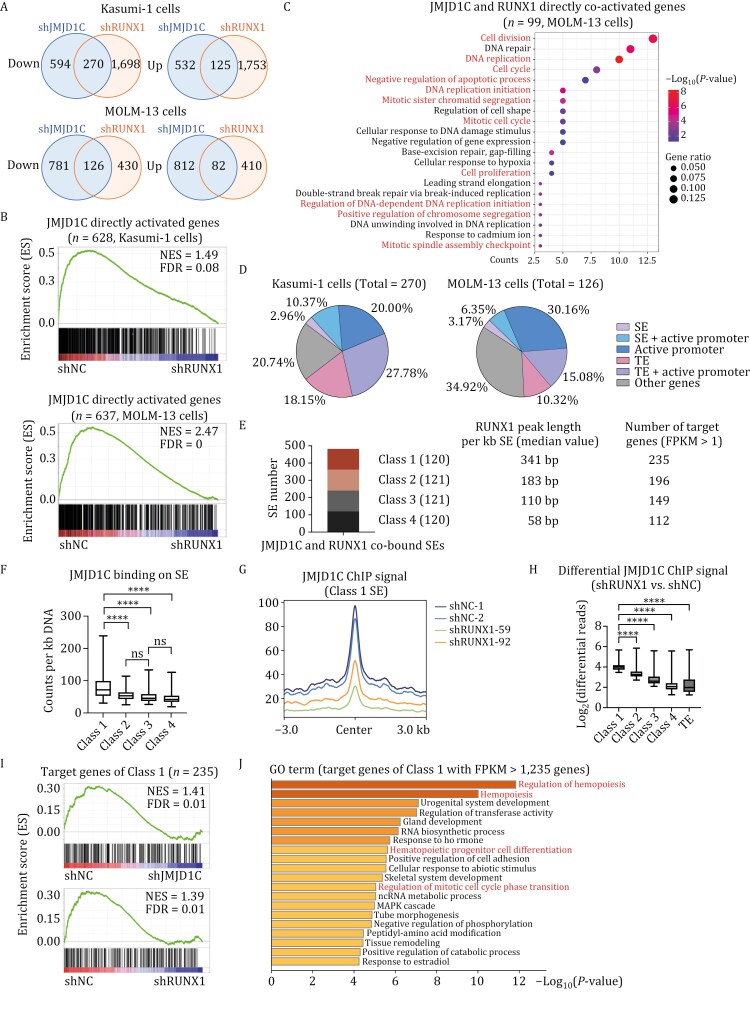
JMJD1C and RUNX1 are enriched on SEs to regulate the leukemic transcriptional program. (A) Venn diagrams showing the overlap of downregulated or upregulated genes in JMJD1C or RUNX1 depleted cells from RNA-seq data in Kasumi-1 (upper panel) and MOLM-13 (lower panel) cells. Differentially expressed genes are genes that changed by 1.5-fold, with FDR < 0.05 upon JMJD1C or RUNX1 depletion. (B) GSEA analyses to determine the enrichment of JMJD1C directly-activated genes in Kasumi-1 (upper panel) and MOLM-13 cells (lower panel). JMJD1C directly-activated genes were defined as genes down-regulated by 1.5-fold, with FDR < 0.05 upon JMJD1C KD, and have nearby JMJD1C and RUNX1 peaks. (C) GO analysis of JMJD1C and RUNX1 directly co-activated genes in MOLM-13 cells. JMJD1C and RUNX1 directly co-activated genes were defined as genes down-regulated by 1.5-fold, with FDR < 0.05 from RNA-seq data upon JMJD1C KD and RUNX1 KD, and have nearby JMJD1C and RUNX1 peaks in MOLM-13 cells. (D) Pie charts depicting JMJD1C and RUNX1 co-activated genes overlapped with target genes of JMJD1C and RUNX1 co-bound active promoters, SEs, and TEs in Kasumi-1 (left panel) and MOLM-13 (right panel) cells. Active promoters were defined as regions within 2.5 kb of TSSs overlapped with H3K27ac peaks. Gene percentages of each group are indicated. (E) Bar plots to show the SE numbers of each class of SEs. The median length of RUNX1 ChIP-seq peaks per kb DNA at each class of SEs and the numbers of potential target genes (with FPKM > 1) for each class of SEs were also shown. (F) Boxplots to compare the counts of JMJD1C ChIP-seq reads per kb DNA at four classes of SEs. The lines indicate median values. *P*-values were determined using ANOVA followed by Dunn’s multiple comparison test, ****P < 0.0001, ns represents not significant. (G) Metaplot to show the changes of JMJD1C ChIP signals upon RUNX1 KD on SEs of Class 1. The normalized JMJD1C ChIP signal values were also used to generate the boxplots, as shown in Fig. 6H. (H) Boxplots to display the log_2_-transformed values of the differential JMJD1C ChIP signal upon RUNX1 KD. The lines indicate median values. *P*-values were determined using ANOVA followed by Kruskal–Wallis’s multiple comparison test, ****P < 0.0001. (I) GSEA analyses to determine the enrichment of potential target genes of Class 1 in RNA-seq data (shJMJD1C vs. shNC, upper panel and shRUNX1 vs. shNC, lower panel) in MOLM-13 cells. (J) GO analysis of potential target genes of Class 1 SEs.

Next, we set out to explore whether JMJD1C and RUNX1 co-regulate target gene expression. To this end, we performed RNA-seq analyses in Kasumi-1 and MOLM-13 cells treated with JMJD1C, RUNX1, or control shRNAs. We first identified differentially expressed genes that are either down- or up-regulated upon JMJD1C or RUNX1 depletion (shJMJD1C vs. shNC or shRUNX1 vs. shNC) by 1.5-fold, with FDR < 0.05. Then, we overlapped the down- or up-regulated genes when JMJD1C or RUNX1 was depleted. Venn diagram analyses revealed a greater number of genes that are concurrently activated by JMJD1C and RUNX1 compared to those that are simultaneously repressed by these two factors (270 vs. 125 genes in Kasumi-1 cells and 126 vs. 82 genes in MOLM-13 cells) (Fig. 6A), suggesting that JMJD1C and RUNX1 preferentially activate genes together. We next further examined the correlation between genes activated by JMJD1C and by RUNX1. GSEA analyses showed that JMJD1C-activated genes are preferentially down-regulated in the RUNX1 KD group (shRUNX1 vs. shNC) in both Kasumi-1 and MOLM-13 cells (Fig. S9A), while JMJD1C-repressed genes did not show significant preference upon RUNX1 depletion (Fig. S9B). These results suggest that JMJD1C preferentially co-activates gene expression with RUNX1. Furthermore, the same correlation was confirmed with JMJD1C directly-activated genes (genes down-regulated by JMJD1C KD and directly co-bound by JMJD1C and RUNX1) (Fig. 6B). Thus, JMJD1C and RUNX1 predominantly co-activate target gene expression.

Next, we examined the related biological processes of JMJD1C and RUNX1 directly co-upregulated genes. We first defined genes directly co-activated by JMJD1C and RUNX1 as genes down-regulated in both JMJD1C KD and RUNX1 KD, and directly bound by both JMJD1C and RUNX1. GO analyses indicate that the JMJD1C and RUNX1 directly co-activated genes in MOLM-13 cells are involved in the cell cycle, DNA replication, and cell division processes (Fig. 6C), all of which are necessary to maintain the hyper-proliferation features and differentiation defects of leukemia cells. Interestingly, in Kasumi-1 cells, GO analysis showed the enrichment of cell differentiation, proliferation, adhesion, and metabolic processes such as one-carbon metabolism and insulin-stimulated cellular response (Fig. S9C), whose alterations have been shown to contribute to AML growth and maintenance (Pikman et al., 2016; Pulikkottil et al., 2022). Moreover, several selected genes that were co-activated by JMJD1C and RUNX1 in Kasumi-1 and MOLM-13 cells (Fig. S9D and S9E) were also up-regulated by both JMJD1C and RUNX1 in other types of AML cells including THP-1 and HL-60 cells (Fig. S9F).

Next, we set out to investigate how much JMJD1C and RUNX1 co-activated genes (Fig. 6A) are regulated by JMJD1C and RUNX1 co-bound active cis-regulatory elements, such as SEs, TEs, and active promoters. Pie charts indicate that the majority (over 65%) of JMJD1C and RUNX1 co-activated genes are bound by both proteins at active cis-regulatory elements, indicating that most of these genes are directly regulated by JMJD1C and RUNX1 (Fig. 6D). The above findings demonstrate that JMJD1C and RUNX1 co-activate target genes expression to maintain leukemia cell growth, survival, and abnormal metabolic activities by binding to promoters and distal enhancers of target genes.

### JMJD1C is enriched on SEs with dense RUNX1 binding to activate gene expression

Since LLPS of transcription regulators has been shown to facilitate the assembly and function of SEs (Hu et al., 2023; Li et al., 2023; Sabari et al., 2018; Wang et al., 2019), we next wanted to examine whether the LLPS ability of JMJD1C regulates the function of SEs and thus controls the RUNX1-regulated gene expression program in AML. We also wanted to see if a higher local concentration of JMJD1C is more likely to facilitate target gene expression. First of all, we analyzed 482 SEs co-bound by JMJD1C and RUNX1, dividing these SEs into four quartiles based on RUNX1 binding density. We considered two methods for defining RUNX1 binding density: RUNX1 ChIP-seq reads (Fig. S9G, left panel) and RUNX1 ChIP-seq peak length (Fig. S9G, right panel) per kilobase of super-enhancer (kb SE). We opted for the classification based on RUNX1 peak length, as it aligns more closely with our hypothesis that denser RUNX1 binding sites could potentially recruit more JMJD1C to form larger condensates that cover closely-situated RUNX1 peaks through the LLPS ability of JMJD1C on SEs. Accordingly, we classified SEs into Classes 1–4 extending from the most to the least dense RUNX1 binding (Fig. 6E). Consistent with our hypothesis, the top quartile (Class 1) SEs with the densest binding of RUNX1 also showed significantly higher levels of JMJD1C ChIP signals per kb DNA than did the other three classes, while there was no significant difference in JMJD1C enrichment among Classes 2, 3, and 4 (Fig. 6F). These results indicate that JMJD1C recruitment to chromatin is increased in a nonlinear manner, and only when RUNX1 chromatin binding reaches a certain threshold of density is JMJD1C recruitment to chromatin further strengthened by its self-interacting LLPS capability. This is consistent with the fact that LLPS ability is concentration-dependent. Additionally, we compared the decrease of JMJD1C occupancy upon RUNX1 KD at Class 1 SEs vs. Class 2–4 SEs or TEs. The decrease in JMJD1C ChIP-seq signal upon RUNX1 KD is indeed most prominent at Class 1 SEs, particularly in the region flanking JMJD1C peak center (Figs. 6G, 6H and S9H), where JMJD1C protein possibly mainly rely on phase separation ability to bind to chromatin.

We next examined whether the significant enrichment of JMJD1C on Class 1 SEs plays a role in RUNX1-regulated gene expression. First, we identified potential target genes of each class of SEs by finding coding genes within 50 kb of each SE (Fig. 6E). Then, we examined the expression changes of these genes for each SE class upon JMJD1C or RUNX1 KD. GSEA analyses showed that JMJD1C and RUNX1 predominantly activate the expression of genes associated with Class 1 SEs (Fig. 6I), and show no similar preference for the genes in Class 2–4 (Fig. S9I–K). Moreover, GO analysis showed that the genes regulated by Class1 SEs are significantly enriched for leukemic programs such as hemopoiesis regulation, cell cycle, and hematopoietic progenitor cell differentiation (Fig. 6J), indicating that Class 1 SEs are key enhancers for AML survival. The *CEBPA* gene locus was taken as an example to show the enrichment of JMJD1C and RUNX1 at Class 1 SEs (Fig. S9L). These findings indicate that JMJD1C and RUNX1 predominantly co-activate target gene expression by regulating active cis-regulatory elements. Notably, JMJD1C forms condensate at super-enhancers with dense RUNX1 binding sites, thereby contributing to leukemic gene activation and leukemogenesis.

### JMJD1C and RUNX1 are essential for enhancer–promoter interactions

**Figure 7.**
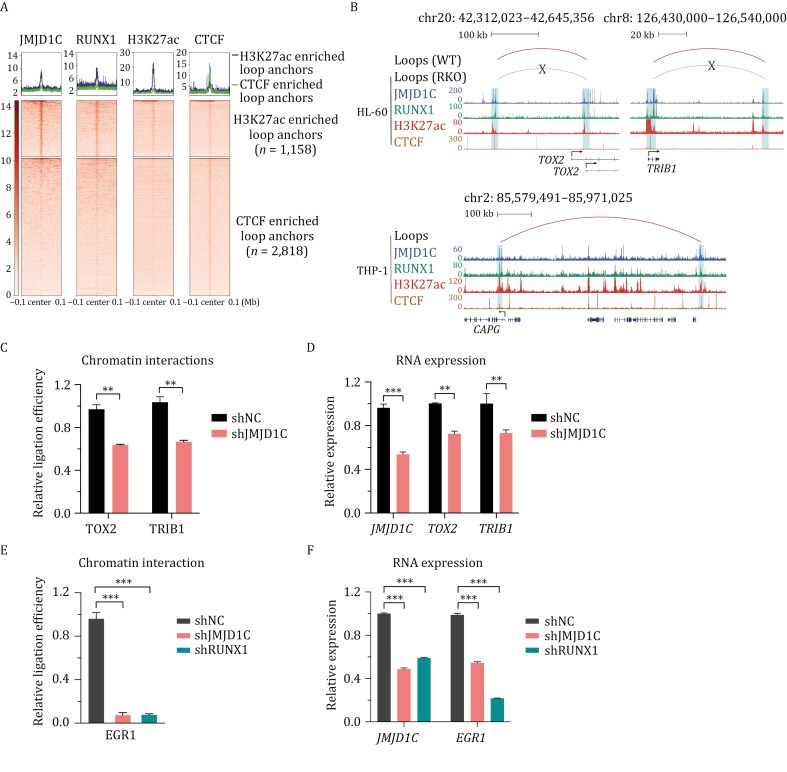
JMJD1C and RUNX1 mediate enhancer–promoter interactions. (A) Heatmaps showing ChIP-seq reads for JMJD1C, RUNX1, H3K27ac, and CTCF, rank-ordered from high to low by JMJD1C occupancy centered at the loop anchors within a ±100 kb genomic region. Color density reflects read density. (B) Representative regions of JMJD1C and RUNX1 co-bound chromatin loops in HL-60 (upper panel) and THP-1 (lower panel) cells. JMJD1C and RUNX1 co-bound loops around *TOX2, TRIB1,* and *CAPG* gene promoters are presented in the loop track. Track names are indicated on left, and RKO represents RUNX1 knockout. Gene names are shown below the snapshots. (C) 3C-qPCR analyses showing interactions of gene promoters and their distal enhancers in HL-60 cells treated with either control shRNA or JMJD1C shRNA. Data are presented as mean ± SD. *P*-values were determined using unpaired two-tailed Student’s *t*-test, **P < 0.01. Black, control shRNA; red, JMJD1C shRNA. (D) RT-qPCR analyses of RNA levels in HL-60 cells treated with either control shRNA or JMJD1C shRNA. Data are presented as mean ± SD. *P*-values were determined using unpaired two-tailed Student’s *t*-test, ***P < 0.001, **P < 0.01. Black, control shRNA; red, JMJD1C shRNA. (E) 3C-qPCR analyses showing the interaction of *EGR1* gene promoter and distal enhancer (the region overlapping *FAM53C* gene locus indicated by red arrow in Fig. S10E) in MOLM-13 cells treated with either control shRNA, JMJD1C shRNA or RUNX1 shRNA. Data are presented as mean ± SD. *P*-values were determined using unpaired two-tailed Student’s *t*-test, ***P < 0.001. Black, control shRNA; red, JMJD1C shRNA; green, RUNX1 shRNA. (F) RT-qPCR analyses of RNA levels in MOLM-13 cells treated with either control shRNA, JMJD1C shRNA or RUNX1 shRNA. Data are presented as mean ± SD. *P*-values were determined using unpaired two-tailed Student’s *t*-test, ***P < 0.001. Black, control shRNA; red, JMJD1C shRNA; green, RUNX1 shRNA.

Since JMJD1C and RUNX1 co-occupied genomic regions are mostly distributed at active promoter and enhancer regions (Figs. 2D and S3H), we hypothesized that JMJD1C self-interaction enables spatial proximity of otherwise far away enhancers and gene promoters, thereby activating the transcription of target genes. To examine this hypothesis, we analyzed public Hi-C datasets (Ochi et al., 2020; Phanstiel et al., 2017) combined with JMJD1C, RUNX1, CTCF, and H3K27ac ChIP-seq datasets in HL-60 and THP-1 cells. Chromatin loops identified by Ochi et al. (2020) in HL-60 cells and Phanstiel et al. (2017) in THP-1 cells were used for the following analyses. CTCF–CTCF interactions play a crucial role in forming chromosome loop structures but only occasionally engage directly in enhancer–promoter contacts (Phillips and Corces, 2009). In contrast, the active enhancer mark (H3K27ac) at anchors of enhancer–promoter contacts correlates with transcriptional activation (Rubin et al., 2017). Next, we aimed to unbiasedly examine whether JMJD1C and RUNX1 are enriched at CTCF-enriched or H3K27ac-enriched loop anchors. Our analysis of JMJD1C and RUNX1 ChIP signals at loop anchors revealed that both JMJD1C and RUNX1 are significantly enriched at H3K27ac-enriched loop anchors but not at CTCF-enriched loop anchors (Figs. 7A and S10A), implying that they may regulate enhancer-involved three-dimensional chromatin structures in leukemia cells. Consistent with this observation, the RUNX motif was enriched in the accessible regions of H3K27ac-enriched loop anchors (Fig. S10B). As three examples, chromatin loops around *TOX2, TRIB1,* and *CAPG* gene promoters are enriched with JMJD1C and RUNX1 binding on both ends in HL-60 and THP-1 cells (Fig. 7B, purple loops).

To further confirm that JMJD1C and RUNX1 are involved in mediating chromatin interactions, we monitored the chromatin interaction changes when JMJD1C or RUNX1 is depleted. We first analyzed published Hi-C data in RUNX1 knockout HL-60 cells (Ochi et al., 2020) and found that 70% of the RUNX1-regulated loops on RUNX1-activated genes (identified by RUNX1 KO) were lost upon RUNX1 KO (Fig. S10C). Next, we want to confirm whether JMJD1C is involved in RUNX1-regulated chromatin loops. Chromatin interactions around *TOX2* and *TRIB1* gene promoters were chosen for the following experiments (Fig. 7B, upper panel). We knocked down JMJD1C with shRNA, performed 3C-qPCR, and found that JMJD1C is also required for chromatin interactions at these loci (Fig. 7C). qPCR results also confirmed that JMJD1C regulates *TOX2* and *TRIB1* expression in HL-60 cells (Fig. 7D). We next wanted to confirm that JMJD1C and RUNX1 also regulate chromatin interactions around *TOX2* and *TRIB1* gene promoters in MOLM-13 cells. However, as these two genes are not significantly downregulated in MOLM-13 cells (Fig. S10D), we took the *EGR1* gene (which is significantly co-activated by JMJD1C and RUNX1) as another example in MOLM-13 cells. JMJD1C and RUNX1 were similarly found to orchestrate chromatin interactions of the *EGR1* promoter with a distal enhancer (a region overlapping the *FAM53C* gene locus, identified in the 4C-seq data shown in Fig. S10E) to activate *EGR1* gene expression in MOLM-13 cells (Fig. 7E and 7F). Notably, the protein levels of the chromatin looping factor LDB1 (Deng et al., 2014) did not change upon JMJD1C KD (Fig. S10F–I). These results indicate that JMJD1C and RUNX1 are both indispensable for mediating chromatin loop formation of leukemic genes and thus for activating their transcription in AML cells.

Taken together, our findings illustrate that the LLPS and RUNX1 interaction abilities of the JMJD1C N-terminal region are both indispensable for JMJD1C-mediated recruitment of RUNX1 and DNA to its condensates. The dual functions of the newly identified N-terminal region may constitute the underlying mechanism for JMJD1C- and RUNX1-dependent chromatin interactions and target gene expression in maintaining the survival of multiple types of AML cells (Fig. S10J).

## Discussion

AML is a highly heterogeneous malignancy with numerous types of genetic mutations and potentially different downstream transcriptional programs, such that the identification of a universal transcriptional regulatory mechanism that governs leukemic gene expression is of great clinical significance. We previously showed that the chromatin-modulating cofactor JMJD1C is universally required for the survival of AML cells with different genetic backgrounds (Chen et al., 2015), but how JMJD1C regulates the leukemic programs of various AML cells has been unclear. In this study, we demonstrate that the noncatalytic N-terminal region of JMJD1C has two functions. The first is to interact with the highly expressed, oncogenic transcription factor RUNX1, which mediates JMJD1C recruitment, and the second is the formation of condensates by self-interaction. These functionalities of the JMJD1C N-terminus, in turn, mediate enhancer–promoter interactions of chromatin regions enriched in both JMJD1C and RUNX1 and thereby activate leukemic gene expression. Our study provides insights into the molecular mechanism by which JMJD1C regulates gene expression and reveals a potentially general transcriptional regulation mechanism shared by different types of leukemia.

Since most *de novo* AML cases have been identified as having at least one driver mutation (Cancer Genome Atlas Research Network et al., 2013; Papaemmanuil et al., 2016), mutation-specific AML dependencies have been actively studied. For example, the H3K79 methyl transferase DOT1L and Menin, which interacts with MLL in conjunction with the H3K36 me2/3 reader LEDGF, have been shown to be especially important for MLL-AF9 leukemia (Bernt et al., 2011; Daigle et al., 2011; Lin et al., 2023). Despite the importance of identifying as many potential dependencies as possible for each type of leukemia, due to the development of drug resistance and cost-effectiveness, it would be of immense importance to develop a common targeted therapy for different types of AML. JMJD1C or the JMJD1C/RUNX1 interaction are such candidates for targeted therapies as JMJD1C is required for the survival of various leukemias but not for HSC homeostasis (Zhu et al., 2016).

Studies of RUNX1 have established its function as a master regulator in the specification and development of the definitive hematopoietic stem cell. Although previous studies have suggested that the leukemic fusion protein AML1-ETO blocks differentiation by dysregulating RUNX1 target genes, recent data actually suggest a positive role for RUNX1 in leukemia (Goyama et al., 2013). The RUNX1 requirement for leukemias with CBFβ and MLL translocations further supports the idea that RUNX1 may actually promote the growth of many types of leukemia cells (Patel et al., 2012; Schnittger et al., 2011; Tang et al., 2009). Indeed, our studies identify a universal requirement for the JMJD1C-RUNX1 axis in multiple AMLs. One remaining question is how RUNX1 functions in combination with other key transcription factors in these various types of leukemia. RUNX1 works in functional complexes with many TFs and cofactors such as TAL1, LYL1, and LDB1 in hematopoietic stem cells or progenitor cells (Wilson et al., 2010). In AML1-ETO leukemia, genomic data have suggested a functional complex among RUNX1, LDB1/LMO2, and HEB (Ptasinska et al., 2019). It is possible that RUNX1 could form a complex with these transcription factors, similar to the complex observed in HSPC or in t(8;21) leukemia, in multiple types of leukemia, thereby regulating key genes important for self-renewal and proliferation.

The communication between promoters and distal cis-regulatory elements and associated factors is essential for transcriptional control both in normal development and in pathologies that include cancer. Many TFs or leukemic fusion TFs have been demonstrated to mediate chromatin interactions (Ahn et al., 2021; Di Giammartino et al., 2019; Magli et al., 2019; Monahan et al., 2019; Ptasinska et al., 2019). In this regard, a recent study showed a functional interaction between RUNX1 and the cohesin component STAG2 that regulates enhancer–promoter interactions and transcription in hematopoiesis (Ochi et al., 2020). That study found that the chromatin loop-promoting RUNX1 binds to loop anchors with high H3K27ac, rather than CTCF-high loop anchors. Similar to the previous work, we found that JMJD1C and RUNX1 also co-bind anchor sites enriched with high H3K27ac. It remains possible that STAG2 is also enriched in these sites, and that the herein demonstrated ability of JMJD1C to form condensates promotes and stabilizes chromatin loops. The high level of H3K27ac on RUNX1- and JMJD1C-regulated chromatin interaction sites suggests that p300 might also be incorporated within JMJD1C/RUNX1-containing condensates to maintain high H3K27ac levels and thus facilitate RUNX1-controlled gene expression. Our analysis of published RUNX1 KO Hi-C and RNA-seq data show that RUNX1 KO leads to the loss of loops of the majority of RUNX1 activated genes targeted by loops with anchors bound by RUNX1. Genes activated by RUNX1 through the regulation of chromatin loops are enriched for GO terms related to cancer cell growth and migration, implying that RUNX1 plays a broad role in enhancer–promoter interactions to regulate leukemic gene expression.

Liquid–liquid phase separation is a principle for explaining transcription complex to precisely control the spatial and temporal expression of genes via distal chromatin interactions in living cells. Recent studies show that chromatin factors could form condensates to regulate oncogenic transcriptional program in tumors. For example, Chromobox 2 (CBX2), one subunit of Polycomb repressive complex 1 (PRC1), was shown to form condensates, direct chromatin compaction, and thus repress the expression of PRC1 target genes (Plys et al., 2019; Tatavosian et al., 2019). Another example is UTX (also known as KDM6A), a core intrinsically disordered region (cIDR) of UTX forms phase-separated liquid condensates, and UTX condensation is essential for genome-wide histone modifications, high-order chromatin interactions, and thus tumor suppression (Shi et al., 2021). In our study, although we observed that JMJD1C condensates could incorporate RUNX1 and RUNX1-bound DNA *in vitro*, it remains unclear whether JMJD1C/RUNX1 puncta occur at endogenous target gene loci *in vivo*. Additionally, further investigation is needed to determine whether the 3D contacts mediated by JMJD1C/RUNX1 are a direct result of condensate formation or an indirect effect due to decreased transcription or potential remodeling of the local chromatin environment. Because JMJD1C knockdown causes cell death, rapid degradation systems, such as the dTAG system, would be essential to the immediate effects of JMJD1C loss.

Our study and prior studies (Chong et al., 2022; Song et al., 2022) underscore a crucial distinction between functional and nonfunctional condensates, providing new insights for the functional study of condensates. We found that JMJD1C is enriched at SEs with dense RUNX1 binding, and RUNX1 depletion significantly reduces JMJD1C occupancy at these SEs, indicating that RUNX1 facilitates JMJD1C condensation at SEs. In contrast, we also found that RUNX1 knockdown does not significantly reduce JMJD1C droplet formation, which is consistent with our *in vitro* droplet formation assay indicating that JMJD1C homotypic interactions are sufficient for its intrinsic droplet formation ability. We hypothesize that the RUNX1-independent condensates are likely not associated with SE target genes or chromatin and are not directly linked to SE target gene expression or 3D genome alterations, as the droplet signals do not overlap with DAPI signals. On the other hand, the RUNX1-assisted JMJD1C condensates on SEs may be much smaller, as high-affinity DNA–TF interactions on SEs might alter the shape of the JMJD1C condensates, making them not as visible as the droplets depending on homotypic interactions. We think that these smaller DNA/TF/JMJD1C condensates are functionally related to gene expression regulation. Consistent with our observations, previous studies have similarly shown that large condensates driven by homotypic interactions may not necessarily be functionally relevant (Chong et al., 2022; Song et al., 2022). How these two types of condensates relate to gene regulation requires further investigation.

In summary, our study describes a non-enzymatic function of JMJD1C in AML, as well as an underlying mechanism that involves a direct interaction with RUNX1 and a self-interaction property that leads to formation of a JMJD1C- and RUNX1-containing condensate and long-range genomic interactions that regulate gene expression. Such interactions of highly expressed lineage-determining transcription factors and chromatin-related cofactors may reflect a potentially general mechanism for transcriptional addiction of cancer cells.

## Materials and methods

### Cell culture

Human AML cell lines were cultured in RPMI-1640 medium (Gibco) supplemented with 10% fetal bovine serum (BioInd) and 1% penicillin and streptomycin at 37°C in a 5% CO_2_ atmosphere. HEK-293T cells were cultured in DMEM medium (Gibco) supplemented with 10% fetal bovine serum (Gemini) and 1% penicillin and streptomycin at 37°C in a 5% CO_2_ atmosphere. Sf9 insect cells were cultured with Insect Cell Culture Medium (ESF 921) at 28°C. All cells were validated to be mycoplasma-free.

### Primary AML cell isolation

Bone marrow samples from AML patients who signed consent forms were obtained from the Nuclear Radiation Injury Protection and Treatment Department of Naval Medical University. The proposed studies were approved by the ethics committee (AFHEC036). Mononuclear cells were isolated from other bone marrow components using density gradient centrifugation with Ficoll-Paque PLUS (Cytiva, 17-1440-02). Primary AML cells were then enriched using the CD34 MicroBead Kit (Miltenyi Biotec, 130-046-702).

### Vector construction and stable cell lines generation

To generate the inducible flag-mCherry-RUNX1-expressing plasmids, human *RUNX1B* gene was cloned into pLVX-Tight-hygro vector with a N-terminal flag-mCherry tag. For lentivirus production, 293T cells were transiently transfected with psPAX2 and pMD2.G packaging plasmids and plasmids with genes of interest (GOI), then cultural supernatants containing lentiviruses were collected 48 h and 72 h after transfection. AML cells (MOLM-13, HL-60, Kasumi-1 and THP-1) expressing inducible flag-mCherry-RUNX1 were generated through lentivirus transduction and then selected with hygromycin (300 ng/μL) for 15 days. To generate JMJD1C truncations-expressing plasmids, N-terminal mEGFP-flag-tagged JMJD1C truncations and N-terminal Halo-HA tagged JMJD1C truncations were cloned into pcDNA3.1 vector or pLVX vector separately. To generate RUNX1 expressing plasmids, RUNX1 with a N-terminal HA tag were cloned into pcDNA3.1 vector. All plasmids were sequenced to confirm identity.

### Co-immunoprecipitation (co-IP)

293T cells were transiently transfected with plasmids in 6-well plates. After 48 h of transfection, cells were collected for nuclear extract preparation as previously described (Dignam et al., 1983). Briefly, cells were resuspended in hypotonic buffer A (10 mmol/L KCl, 10 mmol/L HEPES at pH 7.9, 1.5 mmol/L MgCl_2_, 0.5 mmol/L DTT and 0.5 mmol/L PMSF) and homogenized. Nuclei were collected by centrifugation (3,000 ×*g*, 4°C, 10 min). Then, the nuclei were resuspended in two packed cell pellet volumes of buffer C (25% Glycerol, 600 mmol/L NaCl, 0.2 mmol/L EDTA at pH 8.0, 20 mmol/L HEPES at pH 7.9, 1.5 mmol/L MgCl_2_, 0.5 mmol/L DTT and 0.5 mmol/L PMSF), rocked at 4°C for 1 h. Then the supernatant was used for GFP-IP to examine HA-RUNX1 interaction with different JMJD1C truncations or HA-IP to examine the interactions of mEGFP-flag-tagged and Halo-HA-tagged JMJD1C truncations. GST-GFP nanobody (Fridy et al., 2014) was purified from bacteria and immobilized on glutathione beads (Smart-lifesciences, SA008010). Co-IP samples were incubated and washed with BC150 buffer (20 mmol/L HEPES at pH 7.9, 0.2 mmol/L EDTA, 150 mmol/L NaCl, 0.2% Triton X-100, 10% glycerol, 0.5 mmol/L PMSF and 0.5 mmol/L DTT) for 5 times before Western blot. To examine the association of flag-mCherry-RUNX1 protein with endogenous JMJD1C, flag-mCherry-RUNX1 expression in AML cells was induced by doxycycline (1 μg/mL) for 48 h, then ~6 × 10^7^ AML cells were collected for each IP.

To examine the association of endogenous RUNX1 and JMJD1C, ~6 × 10^7^ AML cells were collected for each IP. Protein A/G beads (Smart-lifesciences, SA032005) were equilibrated with BC150 buffer and then subjected for co-IP assay with 1 μL JMJD1C antibody (Chen et al. 2015) at 4°C for 4 h in BC300 buffer (20 mmol/L HEPES at pH 7.9, 0.2 mmol/L EDTA, 300 mmol/L NaCl, 0.2% Triton X-100, 10% glycerol, 0.5 mmol/L PMSF and 0.5 mmol/L DTT). IP samples were washed with BC150 buffer (20 mmol/L HEPES at pH 7.9, 0.2 mmol/L EDTA, 150 mmol/L NaCl, 0.2% Triton X-100, 10% Glycerol, 0.5 mmol/L PMSF and 0.5 mmol/L DTT) for 5 times before Western blot.

### IP-mass spectrometry (MS)

Nuclear extracts of AML cells (NB-4, HL-60, Kasumi-1 and MOLM-13 cells) were prepared as described in co-IP experiments. IP was performed in BC150 (20 mmol/L HEPES at pH 7.9, 150 mmol/L KCl, 0.2 mmol/L EDTA, 10% glycerol, 0.5 mM DTT, 0.5 mmol/L PMSF) plus 0.1% Triton X-100 overnight at 4°C with rotation. Then, beads were washed with BC150 plus 0.2% Triton X-100. Protein on the beads were eluted with 3X Flag peptide (Sigma, F4799) and subjected to SDS-PAGE and silver staining. Lanes from IgG and JMJD1C group were separately analyzed by liquid chromatography coupled with tandem mass spectrometry (LC-MS/MS).

### Cell proliferation assay and colony formation assay

shRNAs targeting genes of interest were cloned into the pLKO-shRNA-puro vector and lentiviruses were prepared as described above. AML cells were infected with shRNA lentivirus in complete medium for 12 h before changed to fresh complete medium. 48 h after infection, shRNA expressing cells (MOLM-13, HL-60, THP-1, Kasumi-1 and MV4-11) were selected with puromycin (2 μg/mL) for 48 h and subjected for cell proliferation assay. Proliferation assays were performed with CellTiter-Glo® 2.0 Assay (G9242, Promega). 36 h after infection, shRNA expressing cells (Kasumi-1, MOLM-13 and MV4-11) were selected with puromycin (2 μg/mL) for another 36 h and subjected for colony formation assay. Colony assays were performed using methylcellulose media (H4230; *STEMCELL Technologies*). For a full list of shRNA sequences, see Table S6. Notably, shRNAs of RUNX1 specifically target the region specific to RUNX1, the C terminus region that is lost in AML1-ETO translocation.

### CD11b staining and Flow cytometry analysis

AML cells (MOLM-13, HL-60, Kasumi-1 and MV4-11) after 4 days of infection with shRNA lentivirus were subjected for staining with CD11b-FITC antibody (Biolegend, 301330, 1:50). Cells were then stained with DAPI (Solarbio, C0065, 1:50) prior to flow cytometry analysis.

### Growth competition assay

sgRNAs that target different domains of JMJD1C were cloned into LRG2.1-sgRNA-GFP (Addgene #108098) vector and packed into lentiviruses. AML cell lines with doxycycline inducible Cas9 expression were infected with lentivirus of sgRNAs and treated with doxycycline (1 μg/mL) after 48 h of infection, which was set as initial time point Day0. Percentages of GFP^+^ cell were measured by Beckman CytoFLEX S or Accuri C6 Plus (BD Biosciences) every 2 or 3 days after initial time point. For a full list of sgRNA sequences, see Table S7. For cutting efficiency detection of sgRNAs, samples were also collected after doxycycline (1 μg/mL) treatment for 3 days and subjected for genomic DNA extraction with FastPure Cell/Tissue DNA Isolation Mini Kit (Vazyme, DC102-01) and qPCR experiments. For a full list of qPCR primer sequences, see Table S8.

### Protein purification

f-JMJD1C, f-JMJD1C truncated proteins (1–757, 748–1,514 and 1,515–2,358) and mEGFP-f-JMJD1C truncated proteins (550–757 and 1–757) were separately cloned into pFastbac1 vector and transfected into Sf9 cells with FuGENE HD transfection reagents (Promega, E2311) to obtain baculovirus. Sf9 cells were then infected with P3 baculovirus in 100 times dilution and collected in 48 h. Then cells were resuspended with BC500 buffer (20 mmol/L HEPES-KOH at pH 7.9, 10% glycerol, 0.2 mmol/L EDTA at pH 8.0, 500 mmol/L KCl, 0.1% NP40, 0.5 mmol/L DTT, 0.5 mmol/L PMSF) and incubated on ice for 15 min, then homogenized by a 15 mL douncer. Lysates were centrifugated at 18,000 ×g, 4°C for 30 min to obtain soluble protein lysates. Strep-Tactin beads (IBA, 2-1208-010) were used for protein purification. Beads were washed and equilibrated in wash buffer (20 mmol/L HEPES-KOH at pH 7.9, 150 mmol/L NaCl, 0.5 mmol/L DTT, 0.5 mmol/L PMSF) for purification, and then proteins were eluted with 10 mmol/L desthiobiotin in elution buffer (20 mmol/L HEPES-KOH at pH 7.9, 150 mmol/L NaCl, 10 mmol/L desthiobiotin, 0.5 mmol/L DTT, 0.5 mmol/L PMSF).

For mEGFP-flag-tagged JMJD1C (1–647) and JMJD1C (1–647)-EWSR1(IDR) truncations, protein coding sequences were cloned into pLVX vector separately and plasmids were transfected into 293T cells. 48 h after transfection, cells were collected and lysed in BC500 buffer. Flag-M2 beads (Sigma, A2220) were used for protein purification. Proteins were eluted with 150 ng/μL 3× Flag peptides (Glbiochem, 056305) in elution buffer (20 mmol/L HEPES-KOH at pH 7.9, 150 mmol/L NaCl, 150 ng/μL 3× Flag peptides, 0.5 mmol/L DTT, 0.5 mmol/L PMSF).

### Sedimentation assay

Purified EGFP-JMJD1C (1–757) proteins (1.5 μmol/L or 6 μmol/L) were incubated for 30 min at room temperature in sedimentation buffer (20 m HEPES-KOH at pH 7.9, 150  mmol/L KCl). Following incubation, the proteins were centrifuged at 13,500 ×g for 15 min at 25°C. The supernatant and pellet were then immediately separated into two tubes. The pellet was thoroughly resuspended in equal volume of sedimentation buffer as the volume of the supernatant fraction. The samples were subsequently analyzed by SDS-PAGE and CBB staining, and band intensities from both the supernatant and mol/L were quantified using ImageJ software.

### *In vitro* binding assay

Full length and truncated f-JMJD1C proteins were purified from Sf9 cells as previously described. f-mCherry and f-mCherry-RUNX1 proteins were purified from 293T cells transiently transfected with pLVX-f-mCherry or pLVX-f-mCherry-RUNX1 plasmids. HA-RUNX1 protein was purified from 293T cells transiently transfected with pcDNA-HA-RUNX1 plasmid. Nuclear extracts of transfected 293T cells expressing f-mCherry, f-mCherry-RUNX1 and HA-RUNX1 were incubated with GST-mCherry nanobody or anti-HA agarose beads in buffer BC500 (20 mmol/L HEPES at pH 7.9, 500 mmol/L NaCl, 0.2 mmol/L EDTA, 10% glycerol, 0.5 mmol/L DTT and 0.5 mmol/L PMSF) plus 1% Triton X-100. Beads were washed and subjected to CBB staining for quantification. Next, beads bound with f-mCherry or f-mCherry-RUNX1 were incubated with f-JMJD1C full length in buffer BC150 (20 mmol/L HEPES at pH 7.9, 150 mmol/L NaCl, 10% glycerol, 0.2 mmol/L EDTA, 0.5 mmol/L DTT and 0.5 mmol/L PMSF) plus 1.5% BSA and 0.1% NP-40. Beads bound with HA-RUNX1 were incubated with f-JMJD1C truncated proteins in buffer BC150 (20 mmol/L HEPES at pH 7.9, 150 mmol/L NaCl, 10% glycerol, 0.2 mmol/L EDTA, 0.5 mmol/L DTT and 0.5 mmol/L PMSF) plus 1.5% BSA and 0.1% NP-40. Then, beads were wash with BC300 (20 mmol/L HEPES at pH 7.9, 300 mmol/L NaCl, 10% glycerol, 0.2 mmol/L EDTA, 0.5 mmol/L DTT and 0.5 mmol/L PMSF) plus 0.1% NP-40. Finally, immunoprecipitated proteins were analyzed by immunoblotting. GST-mCherry nanobody was purified from bacteria and immobilized on glutathione beads (Smart-lifesciences, SA008010).

### Immunofluorescences (IF)

AML cell lines and primary AML cells were cytospun onto poly-L-lysine-coated slides, fixed with 4% paraformaldehyde at room temperature for 15 min, and then permeabilized with 0.5% Triton X-100 for 10 minutes. The cells were blocked with 4% goat serum and 5% BSA in PBS for 2 h. Primary antibodies were applied for overnight staining (anti-JMJD1C, Santa Cruz Biotechnology, sc-101073, 1:10; anti-RUNX1, abcam, ab229482, 1:200 for MOLM-13, 1:100 for MV4-11 and primary AML cells). AlexaFluor secondary antibodies were then incubated with the cells (Alexa Fluor™ Plus 647, Invitrogen, A32728, 1:5,000; Alexa Fluor™ 568, Invitrogen, A11011, 1:5,000). DNA was labeled with Hoechst (Sigma, B2261) at a concentration of 5 μg/mL. Slides were mounted with 10 μL of mounting medium (Southernbiotech, 0100-01). The JMJD1C and RUNX1 antibodies were validated by IF assay using the same procedures following JMJD1C or RUNX1 knockdown.

### Live cell imaging and fluorescence recovery after photobleaching (FRAP)

293T cells expressing the mEGFP-JMJD1C (1–757) construct were seeded on glass bottom plates. Images were collected on 48 h after transfection on a Nikon A1 HD25 Laser Scanning Confocal Microscope (LSCM) equipped with a preheated (37°C) stage, supplemented with humidified air. Droplet fusion images of mEGFP-JMJD1C (1–757) were acquired using a Plan Apo VC 100× Oil objective, and FRAP images were acquired using a Plan Apo λ 100x Oil objective. FRAP assay was performed on Nikon A1 HD25 Confocal microscope with the 488 nm laser. Regions of interest (ROIs) were bleached for 63ms using 30% laser power (488 nm laser) and images were collected every 2 s post-bleaching. Fluorescence intensity was measured using the NIS-Elements software. Post bleach FRAP recovery data was averaged over seven replicates.

### *In vitro* FRAP assay

mEGFP-JMJD1C (1–757) protein (150 nmol/L) was incubated with 10% (*w/v*) PEG-8000 at room temperature for FRAP assay. Images were collected on a Nikon A1 HD25 LSCM. ROIs were bleached for 1 s using 30% laser power (488 nm laser) and images were collected every 3 s post-bleaching. Fluorescence intensity was measured using the NIS-Elements AR analysis software. Post bleach FRAP recovery data was averaged over six replicates. mEGFP-JMJD1C (550–757) protein (2.5 μmol/L) was incubated with 2% (*w/v* PEG-8000 at room temperature for FRAP assay. Images were collected on a Nikon A1 HD25 LSCM. ROIs were bleached for 63 ms using 10% laser power (488 nm laser line) and images were collected every 4 seconds post-bleaching. Fluorescence intensity was measured using the NIS-Elements AR analysis software. Post bleach FRAP recovery data was averaged over nine replicates.

### *In vitro* droplets formation assay

For droplets formation of mEGFP-JMJD1C (1–757) and mEGFP-JMJD1C (550–757) under different concentrations**,** indicated amounts of protein were mixed with PEG-8000 (final 2% *w/v*) and seeded into 384-well glass plates for 30 min incubation at room temperature. Images were taken with Nikon A1 HD25 Confocal microscope with 488 nm laser and Plan Apo λ 100× Oil objective. Pictures were processed with NIS-Elements AR Analysis software. FITC droplet numbers were quantified for each taken view.

For *in vitro* droplets formation assay with mEGFP-JMJD1C truncations, mCherry-RUNX1 and DNA, mEGFP-JMJD1C truncations were first incubated with mCherry-RUNX1, PEG-8000 and DNA fragments with RUNX1 motifs on ice for 30 min, and then for 1 h at room temperature. mEGFP was used as control protein for mEGFP-JMJD1C truncations and mCherry was used as control protein for mCherry-RUNX1. DAPI was used to label DNA at a concentration of 0.2 ng/μL. To reach similar size droplets, we used a higher concentration of mEGFP-JMJD1C (1–647)-EWSR1(IDR) at 0.6 μmol/L and mEGFP-JMJD1C (1–757) at 0.2 μmol/L for imaging. For mEGFP-JMJD1C 1–647 and mEGFP proteins, the concentrations are same with mEGFP-JMJD1C (1–647)-EWSR1(IDR). Images were taken with Nikon A1 HD25 Confocal microscope with the Plan Apo λ 100× Oil objective. Droplets were identified by FITC signals with NIS-Elements analysis software. Area, TRITC and DAPI intensity were measured for every FITC droplet. FITC droplet numbers were quantified for each taken view.

### Droplets formation assay in 293T cells

mEGFP-flag-tagged JMJD1C truncations were transiently transfected into 293T cells to achieve similar expression levels. 24 h after transfection, cells were seeded on glass coverslip. The next day, cells were washed three times with PBS and then fixed with 4% paraformaldehyde for 15 min. After PBS wash for 3 times, the cells were then incubated in PBS with 5 μg/mL Hoechst (Sigma, B2261) for nuclear DNA labeling. With three extra PBS washing, cells were then mounted with 10 μL mounting medium (Southernbiotech, 0100-01). For confocal image processing, nuclei were identified with the 405 nm channel. FITC intensity, nuclei area and droplets number were measured in every mEGFP-fusion protein-expressing nuclei. All images were processed with NIS-Elements Analysis software.

### Live-cell genomic imaging

Microscopy was performed on a Nikon TiE inverted confocal microscope equipped with an Andor iXon Ultra-897 EM-CCD camera and 405 nm, 488 nm, 561 nm, and 642 nm lasers, using the 60× PLAN APO oil objective (NA = 1.40). Images were acquired using Imaris software (version 10.0.1) by time-lapse microscopy with Z stacks at 0.2 μm steps. Live cell imaging conditions were maintained at 37°C with a 5% CO_2_ atmosphere within a humidified chamber. Image processing procedures were executed through Imaris software. Some images were processed using the “Display Adjustment” function within Imaris to mitigate noise, serving visualization purposes only.

### Western blot

Immunoblots were performed using primary antibodies against JMJD1C (Chen et al. 2015) (custom-made, 1:5,000); RUNX1 (Santa Cruz Biotechnology, sc-365644, 1:500); GFP (CST, 2955S, 1:3,000); mCherry (Easybio, BE2026, 1:3,000); Flag-HRP (Sigma, A8592, 1:5,000); HA (Easybio, BE2007, 1:1000; abcam, ab9110, 1:5,000); LDB1 (abcam, ab96799, 1 :2,000); GAPDH (Easybio, BE0024, 1:5,000) and ACTB (Easybio, BE0021, 1:5,000). Membranes were washed for 15 min, incubated with HRP-conjugated secondary antibodies (Easybio, BE0101 or BE0102, 1:10,000) for 1 h at room temperature, and then washed for exposure.

### RT-qPCR

Total RNA was isolated with FastPure Cell/Tissue Total RNA Isolation Kit (Vazyme, RC101-01). Reverse transcription assays were then performed with NovoScript®Plus All-in-one 1st Strand cDNA Synthesis SuperMix (gDNA Purge) (Novoprotein, E047-01B) or HiScript III All-in-one RT supermix perfert for qPCR (Vazyme, R333-01). Quantitative PCR (qPCR) analyses were performed using NovoStart®SYBR qPCR SuperMix plus (Novoprotein, E096-01B) or ChamQ SYBR qPCR Master Mix (without ROX) (Vazyme, Q321-03). *GAPDH* or *ACTB* was served as an internal control. For a full list of qPCR primer sequences, see Table S8.

### RNA-seq

Total RNA was extracted from Kasumi-1 and MOLM-13 cells with TRIZOL reagent (Invitrogen, 15596018). Libraries were constructed with illumina mRNA-seq library prep kit for RNA sample from Kasumi-1 cells. Libraries were constructed using mRNA-seq V3 library prep kit for illumina (Vazyme, NR611) for RNA extracted from MOLM-13 cells. Libraries were sequenced at 50 bp single-end for Kasumi-1 cells or 150 bp paired-end for MOLM-13 cells on the Illumina platform.

### ChIP-seq

ChIP-seq was performed using JMJD1C (Chen et al., 2015) (custome-made) and RUNX1 antibody (abcam, ab23980). AML cells (2–3 × 10^7^ cells per group) were collected and fixed with 1% formaldehyde at room temperature for 10 min. Then cells were resuspended and sonicated in cold cell lysis buffer (50 mmol/L HEPES at pH 7.5, 140 mmol/L NaCl, 0.1% sodium deoxycholate, 1% Triton X-100, 0.1% SDS, 1 mmol/L EDTA, 0.25% sarkosyl, 1× complete protease inhibitor cocktail). Next, chromatin was centrifuged and the supernatants were diluted for immunoprecipitation. Meanwhile, protein A Dynabeads (Invitrogen) were incubated with JMJD1C or RUNX1 antibody in high salt buffer (20 mmol/L Tris-HCl at pH 7.9, 500 mmol/L NaCl, 2 mmol/L EDTA, 1% Triton X-100, 0.1% SDS and 1× complete protease inhibitor cocktail) at 4°C for 3 h with rotation. Then antibody-bound beads were incubated with chromatin at 4°C overnight with rotation. Next, the beads were washed for seven times with high salt buffer and twice with TE buffer (10 mmol/L Tris-HCl at pH 8.0, 1 mmol/L EDTA). DNA was eluted from beads twice at 65°C in 100 μL elution buffer (10 mmol/L Tris-HCl at pH 8.0, 1 mmol/L EDTA, 200 mmol/L NaCl and 1% SDS). Next, eluted DNA was treated with RNase A and proteinase K, and reverse crosslinked at 65°C for 6 h. DNA was then purified using phenol/chloroform extraction and ethanol precipitation. Library construction was performed using End-It DNA End-Repair Kit, Klenow Fragment (3´→ 5´ exo-) and Quick Ligation kit. Libraries of JMJD1C ChIP-seq in AML cells treated with control or RUNX1 shRNAs were sequenced at 2× 150 bp paired-end on the Illumina NovaSeq 6000 platform by Novogene (MOLM-13 cells) or Anoroad (Kasumi-1 cells). RUNX1 ChIP-seq library in untreated MOLM-13 cells were sequenced at 2× 150 bp paired-end on the Illumina NovaSeq 6000 platform by Novogene. Other JMJD1C ChIP-seq libraries in untreated AML cells were sequenced at 50 bp single-end on the Illumina platform.

### 3C-qPCR

MOLM-13 and HL-60 cells (2–5 × 10^6^ cells per group) were fixed with 2% formaldehyde at room temperature for 10 min. Cells were then resuspended in cold cell lysis buffer (10 mmol/L Tris–HCl at pH 8.0, 10 mmol/L NaCl, 0.2% NP-40 and 1× complete protease inhibitor cocktail). Cell lysate was then centrifuged and the supernatants were discarded. The nuclei were then treated with 0.5% SDS at 62°C for 5 min (MOLM-13 cells) or 0.3% SDS at 62°C for 10 min (HL-60 cells), 2% Triton X-100 solution was used to sequester the SDS. Next, for per 1 × 10^5^ cells, the nuclei were digested three rounds in 1 × restriction buffer with 40 Units of *Hin*dIII (NEB, R3104T) at 37°C for 8–12 h each round. Digested samples were reverse crosslinked and subjected to DNA extraction, followed by qPCR to verify digestion efficiency, with undigested DNA served as control. Digested nuclei were then centrifuged and incubated with 1× T4 ligation buffer and 1,200 U T4 DNA ligase at 16°C for 4 h with a 15 s shaking at 900 rpm every 5 min. Next, the nuclei were treated with RNase A and proteinase K for reverse crosslinking at 65°C for 6 h. DNA was then purified using phenol–chloroform–isoamyl alcohol extraction and ethanol precipitation. DNA was further purified with QIAquick PCR Purification Kit (QIAGEN, 28104), followed by qPCR to test ligation efficiency. Primers used for 3C assay are listed in Table S9.

### 4C-seq

4C-seq protocol was modified from a published paper (Matelot and Noordermeer, 2016). Briefly, 1 × 10^7^ MOLM-13 cells were fixed with 2% formaldehyde for 10 min at room temperature. Fixed cells were lysed in cold cell lysis buffer (10 mmol/L Tris–HCl at pH 8.0, 10 mMOL/l NaCl, 0.2% NP-40 and 1× complete protease inhibitor cocktail) for 10 min on ice with occasionally mixing. The nuclei were then centrifuged and treated with 0.5% SDS at 62°C for 5 min. Next, the nuclei were digested with 400 Units DpnII (NEB, R0543L) at 37°C three rounds and each round for 8–12 h. A 1/50 aliquot was removed as an “undigested control” before the restriction enzyme DpnII was added and a 1/50 aliquot was removed as a “digested control” sample after all three rounds of digestion. These control samples were reverse crosslinked and subjected to run on a 1.5% agarose gel to assess the digestion efficiency. Next, the DNA was ligated with T4 DNA ligase (NEB, M0202M) at 16°C for 4 h with 15 s 900 rpm shaking per 5 min. After this, a 1/70 aliquot of the sample was taken to assess the ligation efficiency. Then, samples were reversed crosslinked as above and purified using phenol–chloroform–isoamyl alcohol solution extraction and ethanol precipitation. Dissolved DNA was digested by adding 1× CutSmart buffer, 0.2 mg/mL BSA and 40 Units NalIII (NEB, R0125L) for an overnight 37°C incubation. After this, DNA was ligated by adding 6,700 Units of T4 DNA ligase (NEB, M0202M) and 1× T4 ligation buffer followed by incubation at 16°C for 4 h with 15 s 900 rpm shaking per 5 min. Next, extracted DNA were amplified using PCR and then purified with QIAquick PCR Purification Kit (QIAGEN, 28104) and KAPA DNA beads. The libraries were then sequenced at 2 × 150 bp paired-end on the Illumina NovaSeq 6000 platform by Novogene. Viewpoint specific 4C-seq PCR primers used in this paper are listed in Table S10.

### RNA-seq data processing and differential gene expression analyses

Single-end RNA-seq reads in Kasumi-1 cells were obtained from two biological replicates of control and shRNA knockdown groups and paired-end RNA-seq reads in MOLM-13 cells were obtained from control and separate shRNA knockdown groups. Raw reads were trimmed with fastp v0.20.1 to filter adapters and low-quality reads, then aligned to the human hg19 genome with Hisat2 v2.2.0. Bam files for downstream analyses were obtained with SAMtools v1.7. The counts per gene were calculated using FeatureCounts v2.0.1 under default parameters. Differential genes were identified using R package DESeq2 v1.26.0 with the cutoff set at fold change > 1.5 and FDR < 0.05. Enrichment of GO terms among differential genes were assessed using DAVID v2023q2. Heatmaps of RNA-seq data were plotted with R package pheatmap (v.1.0.12).

### ChIP-seq data processing

Adapters and low-quality bases were trimmed with trim_galore v0.6.4 and filtered reads were mapped to the human hg19 genome with Bowtie2 v2.3.5.1. SAMtools v1.7 was used to further generate bam files. The public ChIP-seq data used in this study were also processed in the same manner. For TFs, peaks were then called using MACS2 v2.1.1 with the narrow-peak mode. For histone marks, peaks were called by MACS2 v2.1.1 using the broad-peak mode. Bigwig files were generated using bamCoverage of deepTools v3.3.0, normalized with RPKM. For JMJD1C ChIP-seq data in RUNX1 depleted cells, differential peaks were identified with cut off as fold change > 1.5 and FDR < 0.05 in MOLM-13 cells, and cut off as fold change > 1.5 in Kasumi-1 cells, by the R package DiffBind v2.14.0. Heatmaps and metaplots were generated using deepTools v3.3.0. R package. ChIPseeker v1.22.1 was used to annotate ChIP-seq peaks and map genomic distribution. The overlapped peaks of JMJD1C and RUNX1 were obtained with Bedtools v2.29.2 with dedault parameters. R package ChIPseeker v1.22.1 was used to calculate the significance of the overlap of JMJD1C and RUNX1 peaks. Enrichment of GO terms among target genes of RUNX1 recruited JMJD1C peaks was analyzed using DAVID v2023q2. Motif analyses were performed with Homer v4.11. Tracks of ChIP-seq data were generated by UCSC.

### 4C-seq data processing

Paired-end 4C-seq reads were trimmed with trim_galore v0.6.4 and filtered reads were mapped to the human hg19 genome with Bowtie2 v2.3.5.1. R package R.4Cker v0.0.0.9000 was used to identify interacting regions with viewpoint.

### Loop classification and motif analyses

Loop anchors were overlapped with CTCF or H3K27ac peak sites using Bedtools v2.29.2 and separately classified into three classes: both loop anchors with ChIP signals, only one loop anchor with ChIP signals and neither loop anchors with ChIP signals. Homer v4.11 was used with default parameters to enrich for the motif of ATAC-seq peaks overlapped with loop anchors in HL-60 cells. Genes with promoters (±3 kb around the TSS) overlapped loop anchors were identified to be associated with loops.

### Super-enhancer definition and annotation

H3K27ac peaks defined with MACS2 v2.1.1 using the broad-peak mode were used for super-enhancer calling in MOLM-13 and Kasumi-1 cells. Specifically, H3K27ac peaks for SE calling were further defined with parameter “-q 1e-9” in MOLM-13 cells. H3K27ac peaks used for HL-60 cells were from a previously published study (Ochi et al., 2020). Super-enhancer calling was then performed using H3K27ac data of MOLM-13, HL-60 and Kasumi-1 cells using the ROSE tool v1.3.1 (Hnisz et al., 2013; Loven et al., 2013; Whyte et al., 2013). Briefly, active enhancers were defined as H3K27ac peaks excluding regions 2.5 kb flanking TSSs. Next, active enhancers within 12.5 kb of one another were stitched together and stitched enhancers were ranked by increasing signals (H3K27ac ChIP-seq signal subtracting signals from input). The cut-off between typical enhancers and SEs was defined on the enrichment profile as the inflection point of the H3K27ac signal intensity versus the concatenated enhancer rank. Target genes of SEs are defined as transcribed genes with the TSS located within 50 kb of the SE boundary, as determined using the ROSE tool v1.3.1 (Hnisz et al., 2013; Loven et al., 2013; Whyte et al., 2013).

### Quantification and statistical analysis

The statistical significance of two-group comparisons was calculated using Excel or GraphPad Prism v9.0.1. Details are described in figure legends. The following annotations apply for all figures: ns, not significant; *, *P* < 0.05; **, *P* < 0.01; ***, *P* < 0.001; ****, *P* < 0.0001. Default settings were used for all GSEA analyses using GSEA (v.4.1.0). All qPCR data are presented as three technical replicates, with the standard deviation depicted by error bars. All high-throughput sequencing data derived from the control and knockdown samples in this study are based on two or three biological replicates.

## Conclusion

JMJD1C facilitates a RUNX1-driven leukemic transcriptional program across various AML subtypes. Notably, the N-terminal region of JMJD1C, rather than its enzymatic domain, is essential for AML cell survival. Specifically, the dual functions of the JMJD1C N-terminus—LLPS and direct interaction with RUNX1—play a critical role in mediating chromatin interactions between distal enhancers and promoters, thereby regulating the expression of key RUNX1-dependent leukemic genes.

## Supplementary data

Supplementary data is available at *Protein & Cell* online https://doi.org/10.1093/procel/pwae059.

pwae059_suppl_Supplementary_Material

pwae059_suppl_Supplementary_Table_S1

pwae059_suppl_Supplementary_Table_S2

pwae059_suppl_Supplementary_Table_S3

pwae059_suppl_Supplementary_Table_S4

pwae059_suppl_Supplementary_Table_S5

pwae059_suppl_Supplementary_Table_S6-S10

## Data Availability

The main data supporting the results of the study are available within the paper and its Supplementary Information. Any additional information required to reanalyze the data reported in this paper is available for research purposes from the corresponding authors upon request. All high-throughput sequencing data generated in this study is deposited at GEO: GSE251729.

## Code availability

This paper does not report original code.
