# Supplementary material for: JMJD1C forms condensate to facilitate a RUNX1-dependent gene expression program shared by multiple types of AML cells
Source: Protein Cell. 2024 Oct 25;16(5):338–64. doi: 10.1093/procel/pwae059 (PMC12120245; doi:10.1093/procel/pwae059)
Supplement: pwae059_suppl_Supplementary_Material [file pwae059_suppl_supplementary_material.pdf]

Supplementary figure legends

Fig. S1. RUNX1 binds with JMJD1C in various AML cells, related to Figure 1.

(A) Motif enrichment analyses of JMJD1C ChIP-seq peaks by HOMER v4.11. *P*-values, percent enrichment of target sequences for TFs, percent of background and best match TFs in MV4-11, CMK, NB-4 and KG-1 cells are shown. *P*-values of motif enrichment were determined using cumulative binomial distributions by HOMER v4.11.

(B) SDS-PAGE and silver staining of proteins immunoprecipitated by IgG and JMJD1C antibody conjugated to protein A/G agarose beads for Kasumi-1 cells. The same samples were used for IP-MS to identify associated transcription factors shown in Figure 1B.

(C) Immunoprecipitation of IgG or JMJD1C antibody with NEs from four tet-on (TO) AML cell lines with doxycycline induced mCherry-RUNX1 to confirm the association of JMJD1C and mCherry-RUNX1. Bound proteins were detected with antibodies shown on left.

(D) SDS-PAGE and CBB staining of purified f-JMJD1C (left panel), mCherry and mCherry-RUNX1 (right panel) proteins used for *in vitro* binding assay shown in Figure 1D.

(E-F) Analyses of JMJD1C (E) and RUNX1 (F) RNA expression levels in AML samples from TCGA database and normal samples from TCGA and GTEx database analyzed with GEPIA (Tang et al. 2017). Sample numbers for each group were listed below the x axis.

(G) Scatterplot of log2(FPKM) values for JMJD1C and RUNX1 derived from RNA-seq data in AML patients with various genetic mutations from the STJUDE database (McLeod et al. 2021).

Fig. S2. RUNX1 is essential for the survival of JMJD1C-dependent AML cells, related to Figure 1.

(A) Assessment of proliferation for HL-60 and Kasumi-1 cells treated with either control shRNA or three separate RUNX1 shRNAs. Data are presented as mean ± SD. *P*-values were determined using unpaired two-tailed Student's t-test, $^{****}P < 0.0001$, $^{**}P < 0.01$.

(B-C) Western blotting of RUNX1 protein in AML cell lines treated with either control shRNA or three separate RUNX1 shRNAs. GAPDH or ACTB serves as internal control.

(D-F) Assessment of colony formation ability of Kasumi-1 (D) and MV4-11 (E) and MOLM-13 (F) cells treated with either a control shRNA lentivirus or three separate RUNX1 shRNA lentiviruses. Colony counts (upper panels) and representative images (lower panels) are shown. Data are presented as mean ± SD. *P*-values were determined using unpaired two-tailed Student's t-test, $^{****}P < 0.0001$.

(G) Flow cytometry indicating the percentages of CD11b-positive cells from RUNX1 KD and control AML cells.

(H) Bar plots showing the FPKM values of differentiation genes in RUNX1 KD and control groups for Kasumi-1 (left panel) and MOLM-13 (right panel) cells.

(I) The CRISPR perturbation data targeting RUNX1 from DepMap.

Fig. S3. RUNX1 recruits JMJD1C to active promoters, TEs and SEs in various AML cells, related to Figure 2.

(A) Venn diagrams showing the number of overlapped peaks from JMJD1C and RUNX1 ChIP-seq in MV4-11, NB-4, KG-1 and CMK cells. *P*-values were calculated by R package ChIPseeker v1.22.1.

(B) Heatmaps of ChIP-seq reads for JMJD1C and RUNX1, rank-ordered from high to low by RUNX1 occupancy centered in a ± 5kb window around the RUNX1 peak center in eight AML cell lines. Color density reflects read density.

(C) ChIP-seq tracks for JMJD1C and RUNX1 at *MYC* gene locus with its promoter bound by JMJD1C and RUNX1. Track names are indicated on left. Gene name is shown below the snapshot.

(D) Genomic distribution of JMJD1C and RUNX1 co-bound regions in each AML cell line.

(E) Distribution of H3K27ac ChIP-seq signals (total reads) across the 7,881 enhancers in MOLM-13 cells. H3K27ac occupancy is not evenly distributed across the enhancer regions, with a subset of enhancers (the 486 super-enhancers) containing exceptionally high amounts of H3K27ac signals. Several genes nearby SEs and the SE ranks are listed.

(F) Distribution of H3K27ac ChIP-seq signals (total reads) across the 12,412 enhancers in HL-60 cells, with a subset of enhancers (the 588 super-enhancers) containing exceptionally high amounts of H3K27ac signals. Several genes nearby SEs and the SE ranks are listed.

(G) Distribution of H3K27ac ChIP-seq signal (total reads) across the 12,619 enhancers in Kasumi-1 cells, a subset of enhancers (the 742 super-enhancers) containing exceptionally high amounts of H3K27ac signal. Several genes nearby SEs and the SE ranks are listed.

(H) Pie chart depicting JMJD1C and RUNX1 co-bound peaks classified by different genomic regions including promoters with or without H3K27ac signal, SEs and TEs in Kasumi-1 cells. Promoters were defined as regions within 2.5kb of transcription start sites (TSS). Peak percentages of each group are indicated.

Fig. S4. JMJD1C is recruited by RUNX1 to genomic loci, related to Figure 2.

(A) Volcano plot showing the JMJD1C ChIP-seq peaks with decreased (blue dots), increased (red dots) or static (grey dots) signals upon RUNX1 KD in Kasumi-1 cells (shRUNX1 *vs.* shNC).

(B) Heatmaps of JMJD1C and RUNX1 ChIP-seq reads for JMJD1C peaks decreased after RUNX1 depletion, rank-ordered from high to low by JMJD1C occupancy of shNC-1 group and centered in a ± 5kb window around the peak center in Kasumi-1 cells. Color density reflects read density. JMJD1C peaks decreased upon RUNX1 KD are further divided into two groups with either strong RUNX1 binding or weak RUNX1 binding, and the two groups were separately plotted. Strong RUNX1 binding: JMJD1C peaks decreased upon RUNX1 KD that overlap with RUNX1 ChIP-seq peaks identified by MACS2 v2.1.1 with parameter "--ratio 1.1";

weak RUNX1 binding: JMJD1C peaks decreased upon RUNX1 KD that does not have RUNX1 ChIP-seq peaks called by MACS2 with parameter "--ratio 1.1".

(C) GO analysis of the nearest genes of JMJD1C peaks that decreased upon RUNX1 depletion (shRUNX1 *vs*. shNC), and overlapped with RUNX1 peaks in Kasumi-1 cells.

(D-E) ChIP-seq tracks for JMJD1C and RUNX1 at *ETV6, MYB, HOXA, ETS1* and *LYN* gene loci showing JMJD1C peaks (shaded) whose size decreased upon RUNX1 KD. The indicated peaks are also bound by RUNX1 in MOLM-13 cells. Track names are indicated on the left. Gene names are shown below the snapshot.

(F) ChIP-seq tracks for JMJD1C and RUNX1 at *ELF4, LYN, FLI1, ETV6 and ETV7* gene loci showing JMJD1C peaks (shaded) whose size decreased upon RUNX1 KD, which are also bound by RUNX1 in Kasumi-1 cells. Track names are indicated on the left. Gene names are shown below the snapshot.

Fig. S5. The N terminus of JMJD1C is critical for survival of multiple AML cells, related to Figure 3.

(A) SDS-PAGE and CBB staining of purified f-JMJD1C(1-757) (left panel), f-JMJD1C(758-1514) and f-JMJD1C(1515-2358) (right panel) protein, which were used for *in vitro* binding assay shown in Figure 3C.

(B) Bar plots showing the fold depletion of CRISPR-Cas9 screening with pooled sgRNAs targeting the JmjC domain of JMJD1C in different AML cell lines.

(C) Diagram showing the procedure of CRISPR-Cas9 domain screening.

(D) Bar plots of growth competition assays showing the changes of GFP+ cells expressing sgRNAs targeting *Rosa*, *PCNA*, 5'-exon, NTR, ZFD and JmjC domain of JMJD1C in HL-60 cells. sgRNA targeting *Rosa* serves as a negative control and sgRNA targeting *PCNA* serves as a positive control. Y-axis represents the GFP percentages of day 12 divided by day 0 after sgRNA expressing cells reach maximal levels of GFP expression, at which point Cas9 was induced with doxycycline. Data are presented as mean ± SD. *P*-values were determined using unpaired two-tailed Student's t-test, ****$P < 0.0001$, ***$P < 0.001$, **$P < 0.01$, *$P < 0.05$, n.s. represents not significant.

(E-H) Bar plots showing the cutting efficiency of sgRNAs used for the growth competition assay in HL-60 (E), MOLM-13 (F), MV4-11 (G) and Kasumi-1 (H) cells. Data are presented as mean ± SD.

Fig. S6. The N-terminal IDR of JMJD1C forms condensates, related to Figure 4.

(A) Graph plotting intrinsic disorder region for JMJD1C. PONDR VSL2 scores are shown on the y axis, and amino acid positions are shown on the x axis.

(B-C) Representative images for immunofluorescence staining of JMJD1C in control and JMJD1C KD groups in MOLM-13 (B, upper panel) and Kasumi-1 cells (C, upper panel) and

quantification of JMJD1C fluorescence intensity normalized to the area of nucleus (lower panels). The scale bar represents 5 μm. *P*-values were determined using ANOVA followed by Dunn's multiple comparison test, ****$P < 0.0001$, ***$P < 0.001$.

(D) Western blotting of the protein levels of JMJD1C truncations in 293T cells transfected with indicated constructs as shown in Figures 4B-C. GAPDH serves as an internal control.

(E) Representative images of mEGFP-JMJD1C(1-757) fusion events in 293T cells. Arrows, fusing droplets. The scale bar represents 5 μm.

(F) SDS-PAGE and CBB staining of purified mEGFP-JMJD1C(1-757) (left panel) and mEGFP-JMJD1C(550-757) (right panel) proteins used for *in vitro* droplets formation and FRAP assays.

(G) Representative SDS-PAGE gel showing the solubility of EGFP-JMJD1C(1-757) in solution. EGFP-JMJD1C(1-757) protein samples at two different concentrations were centrifuged, and equal volumes of the supernatant (S) and pellet (P) fractions were analyzed by CBB staining.

(H) Representative images of mEGFP-JMJD1C(550-757) fusion events *in vitro*. Arrows, fusing droplets. The scale bar represents 5 μm.

(I) Representative images of mEGFP-JMJD1C(550-757) *in vitro* FRAP assay (upper panel) and the quantification (lower panel). Arrows, bleached puncta. The scale bar represents 5 μm. Y-axis represents relative fluorescence intensity, with pre-bleaching signal set as "1". X-axis, time after photobleaching. Data are shown as average relative intensity ± SD (n = 9).

Figure S7. The N-terminal IDR of JMJD1C is functionally promiscuous, related to Figure 4.

(A) Table to summarize the LLPS and RUNX1 interaction abilities of different JMJD1C truncations.

(B) Immunoprecipitation using GFP nanobody-conjugated beads from 293T NEs expressing different mEGFP-f-JMJD1C truncations and HA-RUNX1. Bound proteins were detected with antibodies shown on left. Quantified and normalized values of co-IPed HA-RUNX1 are indicated below each lane. The co-IPed HA-RUNX1 value was determined by the HA signals in the IP group normalized by HA-RUNX1 input group, then further normalized to the bait mEGFP-f-JMJD1C truncations signals. The value in Lane 12 was set as "1".

(C) Representative images of 293T cells transfected with indicated mEGFP-JMJD1C truncations. The scale bar represents 5 μm.

(D) Western blotting of the protein levels of JMJD1C truncations in 293T cells transfected with indicated constructs as shown in Figures S7C. GAPDH serves as an internal control.

(E) Immunoprecipitation using GFP nanobody-conjugated beads from 293T NEs expressing different mEGFP-f-JMJD1C truncations and HA-RUNX1. Bound proteins were detected with antibodies shown on left. Quantified and normalized values of co-IPed HA-RUNX1 are indicated below each lane. The co-IPed HA-RUNX1 value was determined by the HA signals

in the IP group normalized by HA-RUNX1 input group, then further normalized to the bait mEGFP-f-JMJD1C truncations signals. The value in Lane 9 was set as “1”.

(F) Immunoprecipitation of anti-HA agarose beads with 293T NEs expressing mEGFP-f-JMJD1C(1-757) and different Halo-HA-JMJD1C truncations. Bound proteins were detected with antibodies shown on left. Quantified and normalized values of co-IPed mEGFP-f-JMJD1C(1-757) are indicated below each lane. The co-IPed mEGFP-f-JMJD1C(1-757) value was determined by the flag signals in the IP group normalized by mEGFP-f-JMJD1C(1-757) input group, then further normalized to the bait Halo-HA-JMJD1C signals. The value in Lane 7 was set as “1”.

Figure S8. The N-termini of JMJD1C incorporate RUNX1 into the condensates, related to Figure 5.

(A) Representative images for immunofluorescence staining of JMJD1C and RUNX1 in MOLM-13 and MV4-11 cells. The scale bar represents 2 μm.

(B-C) Representative images for immunofluorescence staining of RUNX1 in control and RUNX1 KD groups in MOLM-13 (B, upper panel) and MV4-11 cells (C, upper panel) and quantification of JMJD1C fluorescence intensity normalized to the area of nucleus (lower panels). The scale bars represent 5 μm. *P*-values were determined using Mann-Whitney test, ****$P < 0.0001$.

(D) SDS-PAGE and CBB staining of purified mCherry-RUNX1 (left panel), mEGFP-JMJD1C(1-647) (middle panel) and mEGFP-JMJD1C(1-647)-EWSR1(IDR) (right panel) proteins used for *in vitro* binding assay shown in Figure 5C.

(E) Western blotting of the protein levels of JMJD1C truncations in 293T cells transfected with indicated constructs used in Figure S8F, GAPDH serves as an internal control.

(F) Representative images of 293T cells transfected with mEGFP tagged JMJD1C(1-757) or JMJD1C(1-647)-ESWR1(IDR) (left panel) and quantification of droplets numbers per nucleus of 293T cells (right panel). The scale bar represents 5 μm. *P*-values were determined using ANOVA followed by Dunn’s multiple comparison test, n.s. represents not significant.

Fig. S9. JMJD1C is enriched on SEs with dense RUNX1 binding to activate gene expression, related to Figure 6.

(A) GSEA analyses to determine the enrichment of JMJD1C activated genes (shJMJD1C *vs.* shNC) in RUNX1 KD samples (shRUNX1 *vs.* shNC) in Kasumi-1 (left panel) and MOLM-13 (right panel) cells. JMJD1C activated genes are genes down-regulated upon JMJD1C KD by 1.5-fold, with FDR < 0.05, as shown in Figure 6A.

(B) GSEA analyses to determine the enrichment of JMJD1C repressed genes (shJMJD1C *vs.* shNC) in RUNX1 KD samples (shRUNX1 *vs.* shNC) in Kasumi-1 (left panel) and MOLM-13

(right panel) cells. JMJD1C repressed genes are genes up-regulated upon JMJD1C KD by 1.5-fold, with FDR < 0.05, as shown in Figure 6A.

(C) GO analysis of JMJD1C and RUNX1 directly co-activated genes in Kasumi-1 cells. JMJD1C and RUNX1 directly co-activated genes are defined as described in Figure 6C.

(D-E) Heatmaps showing *Z*-scores of JMJD1C and RUNX1 co-activated genes identified with RNA-seq data from both MOLM-13 and Kasumi-1 cells. *Z*-scores represent the FPKM in control, JMJD1C KD and RUNX1 KD MOLM-13 (D) and Kasumi-1 (E) cells.

(F) RT-qPCR analyses of RNA levels in THP-1 (upper panel) and HL-60 cells (lower panel) treated with either control shRNA, two separate JMJD1C shRNAs or two separate RUNX1 shRNAs. JMJD1C and RUNX1 co-activated genes in both MOLM-13 and Kasumi-1 cells were selected for validation. Data are presented as mean ± SD. *P*-values were determined using unpaired two-tailed Student's t-test, ****P* < 0.001, ***P* < 0.01, **P* < 0.05. Grey, control shRNA; red, JMJD1C shRNAs; green, RUNX1 shRNAs.

(G) A schematic diagram showing the two possible types of RUNX1 binding pattern on SEs.

(H) Metaplots to show the changes of JMJD1C ChIP signals upon RUNX1 KD on TEs and Class2-4 of SEs. The normalized JMJD1C ChIP signal values were also used to generate the boxplots, as shown in Figure 6H.

(I-K) GSEA analyses to determine the enrichment of potential target genes of each class of SEs (Class2, I; Class3, J; Class4, K) in RNA-seq data (shJMJD1C *vs.* shNC, left panels and shRUNX1 *vs.* shNC, right panels) in MOLM-13 cells.

(L) ChIP-seq tracks for JMJD1C (blue), RUNX1 (green) and H3K27ac (red) near *CEBPA* gene locus showing that JMJD1C and RUNX1 are enriched at a Class1 SE. Track names are indicated on left. Gene names are shown below the snapshot.

Fig. S10. JMJD1C and RUNX1 are both required for enhancer-promoter interactions of leukemic genes, related to Figure 7.

(A) Bar plots showing the percentages of loops with anchors overlapped with H3K27ac peaks in HL-60 and THP-1 cells. Y-axis represents the percentages of loops that are bound by H3K27ac at both anchors (Both), one of the two anchors (Either), or neither of the two anchors (Neither).

(B) Motifs enriched at the ATAC-seq peaks overlapped with H3K27ac enriched loop anchors (red boxes in Fig. S10A of HL-60 cells). *P*-values and best match TFs are shown. *P*-values of motif enrichment were determined using cumulative binomial distributions, as applied by Homer v4.11.

(C) Bar plots to show the percentage of lost loops upon RUNX1 knockout, with the total loops defined as those with anchors bound by RUNX1 and targeting RUNX1-activated genes.

(D) Bar plots showing the fold changes of *TOX2* and *TRIB1* RNA expression from RNA-seq

data in MOLM-13 cells upon JMJD1C or RUNX1 KD.

(E) 4C-seq and ChIP-seq tracks at genomic region upstream ~500kb from *EGR1* gene promoter. Shaded gray areas are highly interacted regions with *EGR1* gene promoter and co-bound by JMJD1C and RUNX1. Track names are indicated on left. Gene names are shown below the snapshot.

(F) RT-qPCR analyses of *JMJD1C* and *EGR1* RNA levels in MOLM-13 cells treated with either control shRNA or two separate JMJD1C shRNAs. Data are presented as mean ± SD. *P*-values were determined using unpaired two-tailed Student's t-test, ****$P < 0.0001$.

(G) Western blotting of JMJD1C and LDB1 proteins in MOLM-13 cells treated with either control shRNA or two separate JMJD1C shRNAs. GAPDH serves as an internal control.

(H) RT-qPCR analyses of *JMJD1C*, *TOX2* and *TRIB1* RNA levels in HL-60 cells treated with either control shRNA or two separate JMJD1C shRNAs. Data are presented as mean ± SD. *P*-values were determined using unpaired two-tailed Student's t-test, ****$P < 0.0001$, ***$P < 0.001$, **$P < 0.01$.

(I) Western blotting of JMJD1C and LDB1 proteins in HL-60 cells treated with either control shRNA or two separate JMJD1C shRNAs. ACTB serves as an internal control.

(J) JMJD1C and RUNX1 are universally required for AML cell survival with different genetic backgrounds. Mechanistically, JMJD1C is recruited to active promoters and enhancers by RUNX1 and thus facilitates RUNX1 to activate universal transcriptional program. The N-terminus of JMJD1C, rather than JmjC or zinc finger domains, is generally required for AML cell survival. JMJD1C N-terminal region has dual functions, direct interaction with RUNX1 and phase separation ability. JMJD1C and RUNX1 could mediate chromatin interaction via JMJD1C N-terminus formed droplets. The depletion of JMJD1C or RUNX1 disrupts the enhancer-promoter interaction and dysregulates leukemic gene transcription, leading to AML cell death. The model illustration was created in BioRender. C, Q. (2023) https://BioRender.com/ x65n623.

Figure S1

A

MV4-11 cells

Rank	Motif	P-value	Targets	Background	TF
1		1e-8278	43.39%	10.79%	ETS
2		1e-3865	33.06%	11.53%	RUNX
3		1e-2261	15.70%	4.37%	CEBP
4		1e-2185	13.70%	3.48%	AP-1 (bZIP)
5		1e-1974	9.09%	1.67%	IRF

CMK cells

Rank	Motif	P-value	Targets	Background	TF
1		1e-4083	49.19%	11.39%	GATA
2		1e-2635	56.78%	22.06%	ETS
3		1e-1744	23.53%	5.32%	RUNX
4		1e-207	25.10%	16.80%	BHLHE22
5		1e-128	6.11%	2.94%	AP-1 (bZIP)

NB-4 cells

Rank	Motif	P-value	Targets	Background	TF
1		1e-3506	24.80%	7.68%	ETS
2		1e-2785	27.15%	10.47%	RUNX
3		1e-2720	24.95%	9.20%	CEBP
4		1e-2440	9.02%	1.40%	CTCF
5		1e-1598	8.34%	1.87%	AP-1 (bZIP)

KG-1 cells

Rank	Motif	P-value	Targets	Background	TF
1		1e-6444	59.42%	16.44%	ETS
2		1e-3467	37.96%	10.76%	RUNX
3		1e-565	18.73%	9.37%	GATA
4		1e-330	2.32%	0.36%	CTCF
5		1e-272	17.06%	10.49%	Twist2 (bHLH)

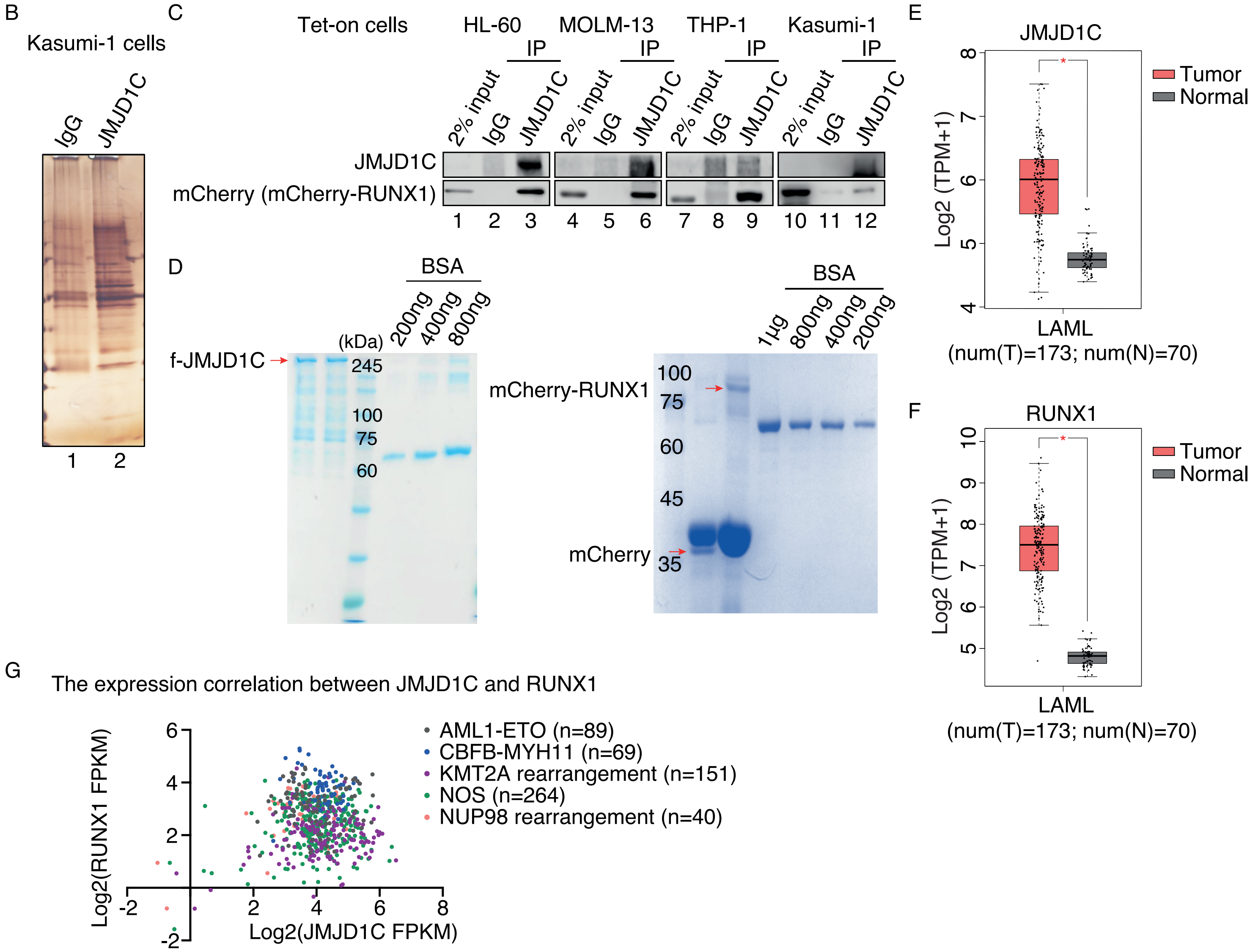

Figure S2

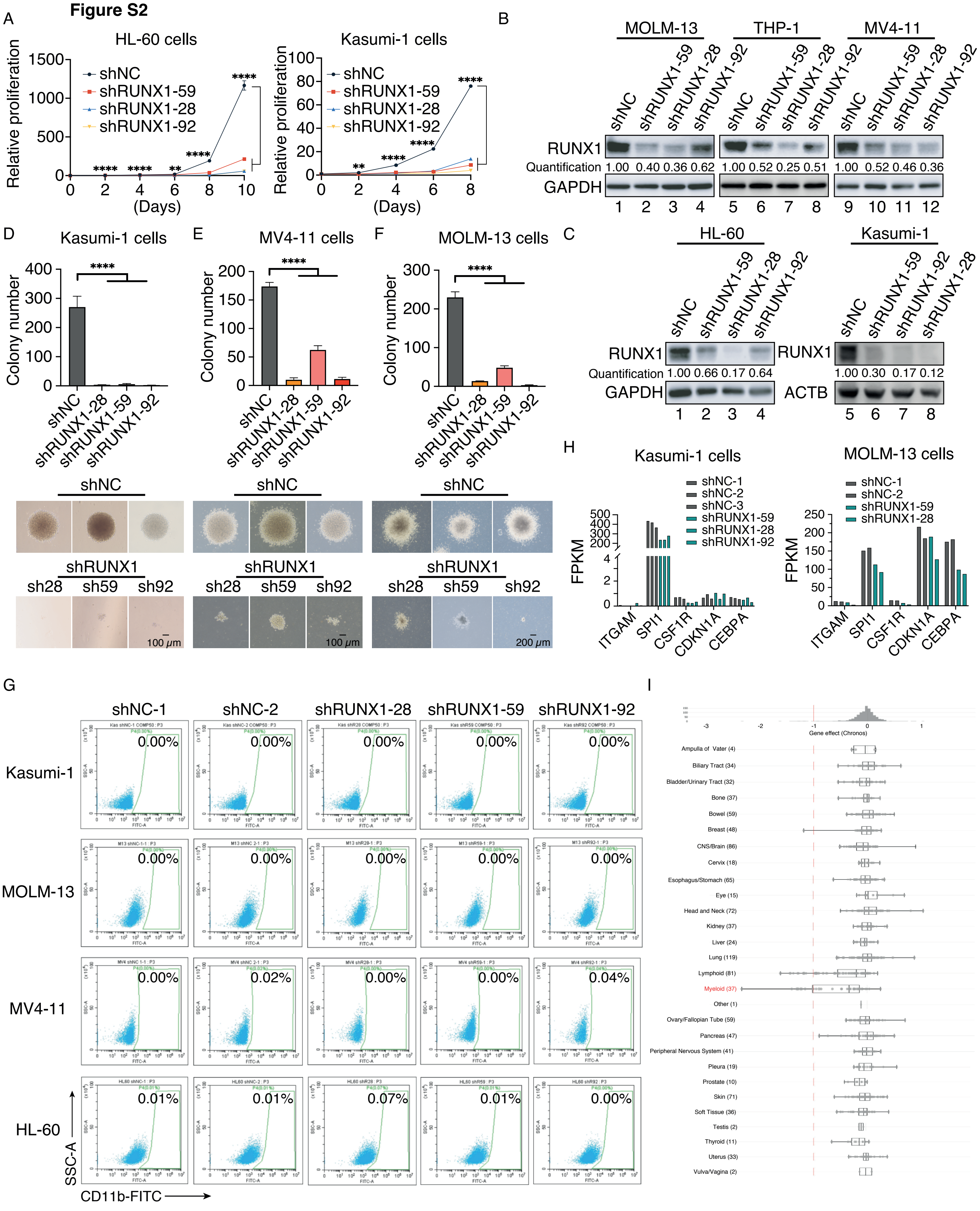

A
HL-60 cells
Kasumi-1 cells
Relative proliferation
(Days)
shNC
shRUNX1-59
shRUNX1-28
shRUNX1-92
B
MOLM-13
THP-1
MV4-11
RUNX1
Quantification 1.00 0.40 0.36 0.62 1.00 0.52 0.25 0.51 1.00 0.52 0.46 0.36
GAPDH
1 2 3 4 5 6 7 8 9 10 11 12
C
HL-60
Kasumi-1
RUNX1
Quantification 1.00 0.66 0.17 0.64
1.00 0.30 0.17 0.12
GAPDH
ACTB
1 2 3 4 5 6 7 8
D
Kasumi-1 cells
E
MV4-11 cells
F
MOLM-13 cells
Colony number
shNC
shRUNX1
sh28 sh59 sh92
100 μm
200 μm
H
Kasumi-1 cells
MOLM-13 cells
FPKM
shNC-1
shNC-2
shNC-3
shRUNX1-59
shRUNX1-28
shRUNX1-92
ITGAM
SPI1
CSF1R
CDKN1A
CEBPA
G
shNC-1
shNC-2
shRUNX1-28
shRUNX1-59
shRUNX1-92
Kasumi-1
MOLM-13
MV4-11
HL-60
0.00% 0.00% 0.00% 0.00% 0.00%
0.00% 0.00% 0.00% 0.00% 0.00%
0.00% 0.02% 0.00% 0.00% 0.04%
0.01% 0.01% 0.07% 0.01% 0.00%
SSC-A
CD11b-FITC
I
Gene effect (Chronos)
Ampulla of Vater (4)
Biliary Tract (34)
Bladder/Urinary Tract (32)
Bone (37)
Bowel (59)
Breast (48)
CNS/Brain (86)
Cervix (18)
Esophagus/Stomach (65)
Eye (15)
Head and Neck (72)
Kidney (37)
Liver (24)
Lung (119)
Lymphoid (81)
Myeloid (37)
Other (1)
Ovary/Fallopian Tube (59)
Pancreas (47)
Peripheral Nervous System (41)
Pleura (19)
Prostate (10)
Skin (71)
Soft Tissue (36)
Testis (2)
Thyroid (11)
Uterus (33)
Vulva/Vagina (2)

Figure S3

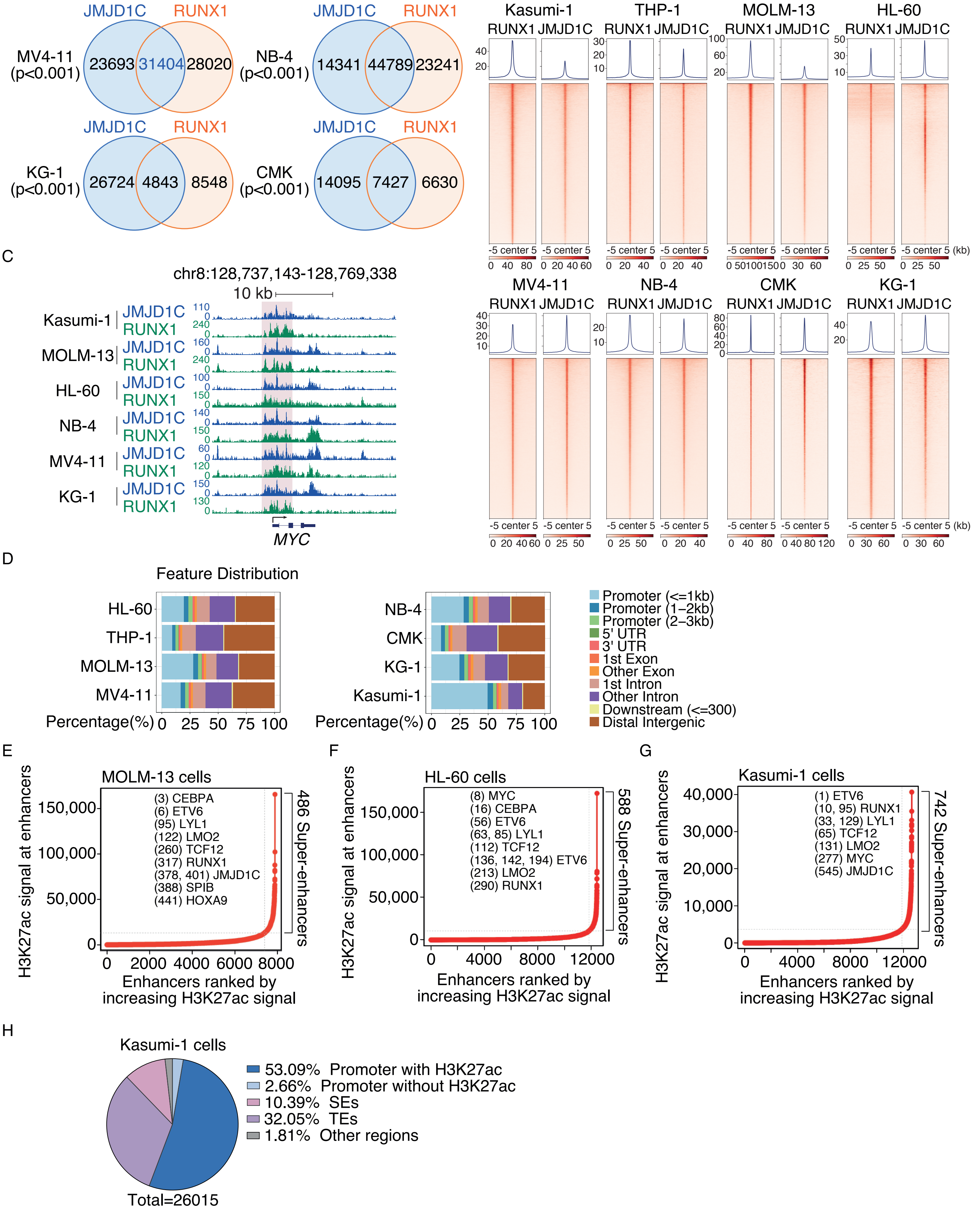
A
JMJD1C
RUNX1
MV4-11
(p<0.001)
23693
31404
28020
NB-4
(p<0.001)
14341
44789
23241
KG-1
(p<0.001)
26724
4843
8548
CMK
(p<0.001)
14095
7427
6630
B
Kasumi-1
THP-1
MOLM-13
HL-60
MV4-11
NB-4
CMK
KG-1
RUNX1
JMJD1C
-5 center 5
(kb)
C
chr8:128,737,143-128,769,338
10 kb
Kasumi-1
MOLM-13
HL-60
NB-4
MV4-11
KG-1
MYC
D
Feature Distribution
HL-60
THP-1
MOLM-13
MV4-11
NB-4
CMK
KG-1
Kasumi-1
Percentage(%)
0 25 50 75 100
Promoter (<=1kb)
Promoter (1–2kb)
Promoter (2–3kb)
5' UTR
3' UTR
1st Exon
Other Exon
1st Intron
Other Intron
Downstream (<=300)
Distal Intergenic
E
MOLM-13 cells
H3K27ac signal at enhancers
(3) CEBPA
(6) ETV6
(95) LYL1
(122) LMO2
(260) TCF12
(317) RUNX1
(378, 401) JMJD1C
(388) SPIB
(441) HOXA9
486 Super-enhancers
Enhancers ranked by increasing H3K27ac signal
F
HL-60 cells
(8) MYC
(16) CEBPA
(56) ETV6
(63, 85) LYL1
(112) TCF12
(136, 142, 194) ETV6
(213) LMO2
(290) RUNX1
588 Super-enhancers
G
Kasumi-1 cells
(1) ETV6
(10, 95) RUNX1
(33, 129) LYL1
(65) TCF12
(131) LMO2
(277) MYC
(545) JMJD1C
742 Super-enhancers
H
Kasumi-1 cells
53.09% Promoter with H3K27ac
2.66% Promoter without H3K27ac
10.39% SEs
32.05% TEs
1.81% Other regions
Total=26015

Figure S4

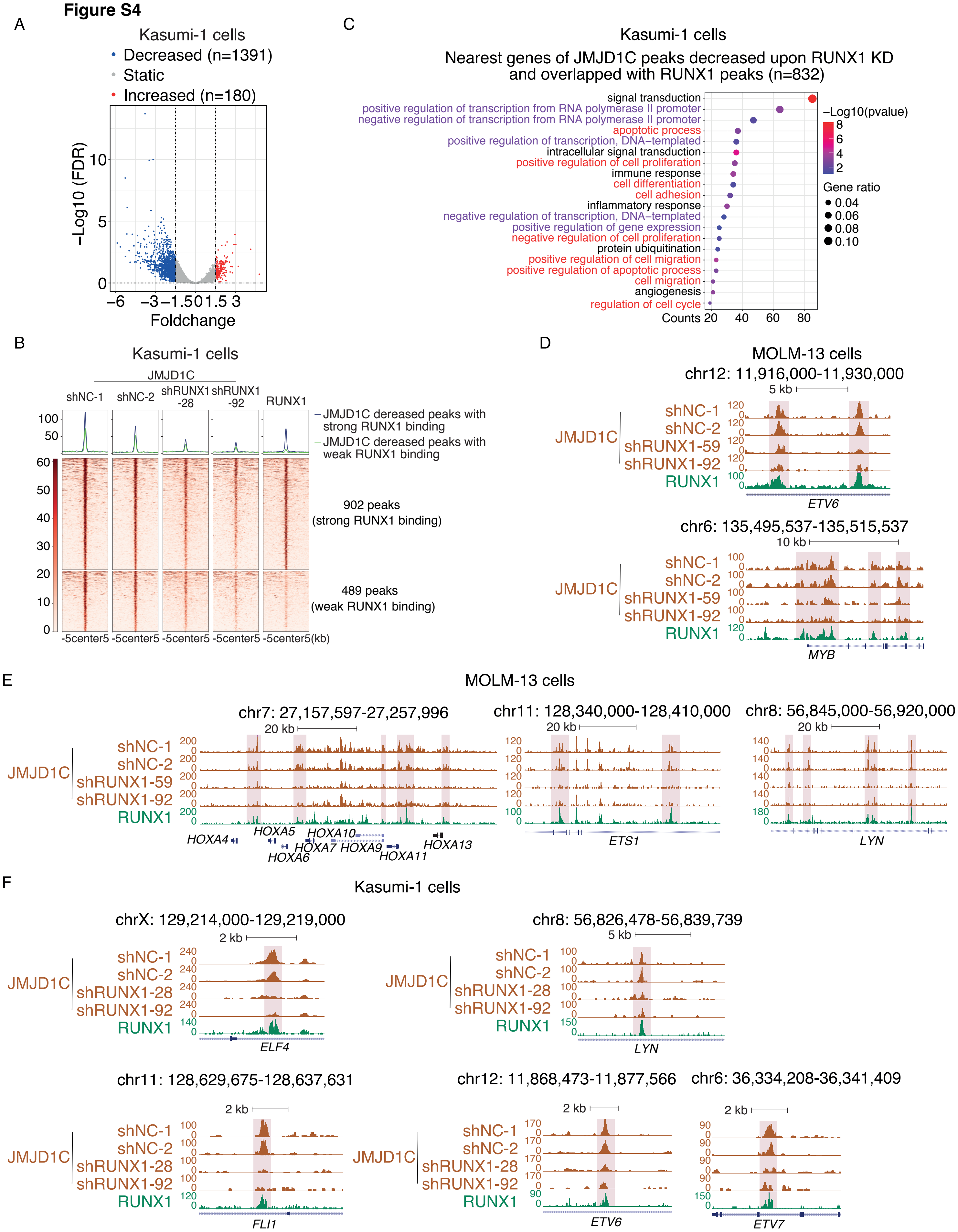
A
Kasumi-1 cells
Decreased (n=1391)
Static
Increased (n=180)
−Log10 (FDR)
Foldchange
C
Kasumi-1 cells
Nearest genes of JMJD1C peaks decreased upon RUNX1 KD
and overlapped with RUNX1 peaks (n=832)
signal transduction
positive regulation of transcription from RNA polymerase II promoter
negative regulation of transcription from RNA polymerase II promoter
apoptotic process
positive regulation of transcription, DNA−templated
intracellular signal transduction
positive regulation of cell proliferation
immune response
cell differentiation
cell adhesion
inflammatory response
negative regulation of transcription, DNA−templated
positive regulation of gene expression
negative regulation of cell proliferation
protein ubiquitination
positive regulation of cell migration
positive regulation of apoptotic process
cell migration
angiogenesis
regulation of cell cycle
Counts
−Log10(pvalue)
Gene ratio
B
Kasumi-1 cells
JMJD1C
shNC-1
shNC-2
shRUNX1 -28
shRUNX1 -92
RUNX1
JMJD1C dereased peaks with strong RUNX1 binding
JMJD1C dereased peaks with weak RUNX1 binding
902 peaks
(strong RUNX1 binding)
489 peaks
(weak RUNX1 binding)
-5center5 -5center5 -5center5 -5center5 -5center5(kb)
D
MOLM-13 cells
chr12: 11,916,000-11,930,000
5 kb
JMJD1C
shNC-1
shNC-2
shRUNX1-59
shRUNX1-92
RUNX1
ETV6
chr6: 135,495,537-135,515,537
10 kb
MYB
E
MOLM-13 cells
chr7: 27,157,597-27,257,996
20 kb
HOXA4
HOXA5
HOXA6
HOXA7
HOXA9
HOXA10
HOXA11
HOXA13
chr11: 128,340,000-128,410,000
ETS1
chr8: 56,845,000-56,920,000
LYN
F
Kasumi-1 cells
chrX: 129,214,000-129,219,000
2 kb
shRUNX1-28
ELF4
chr8: 56,826,478-56,839,739
LYN
chr11: 128,629,675-128,637,631
FLI1
chr12: 11,868,473-11,877,566
ETV6
chr6: 36,334,208-36,341,409
ETV7

Figure S5

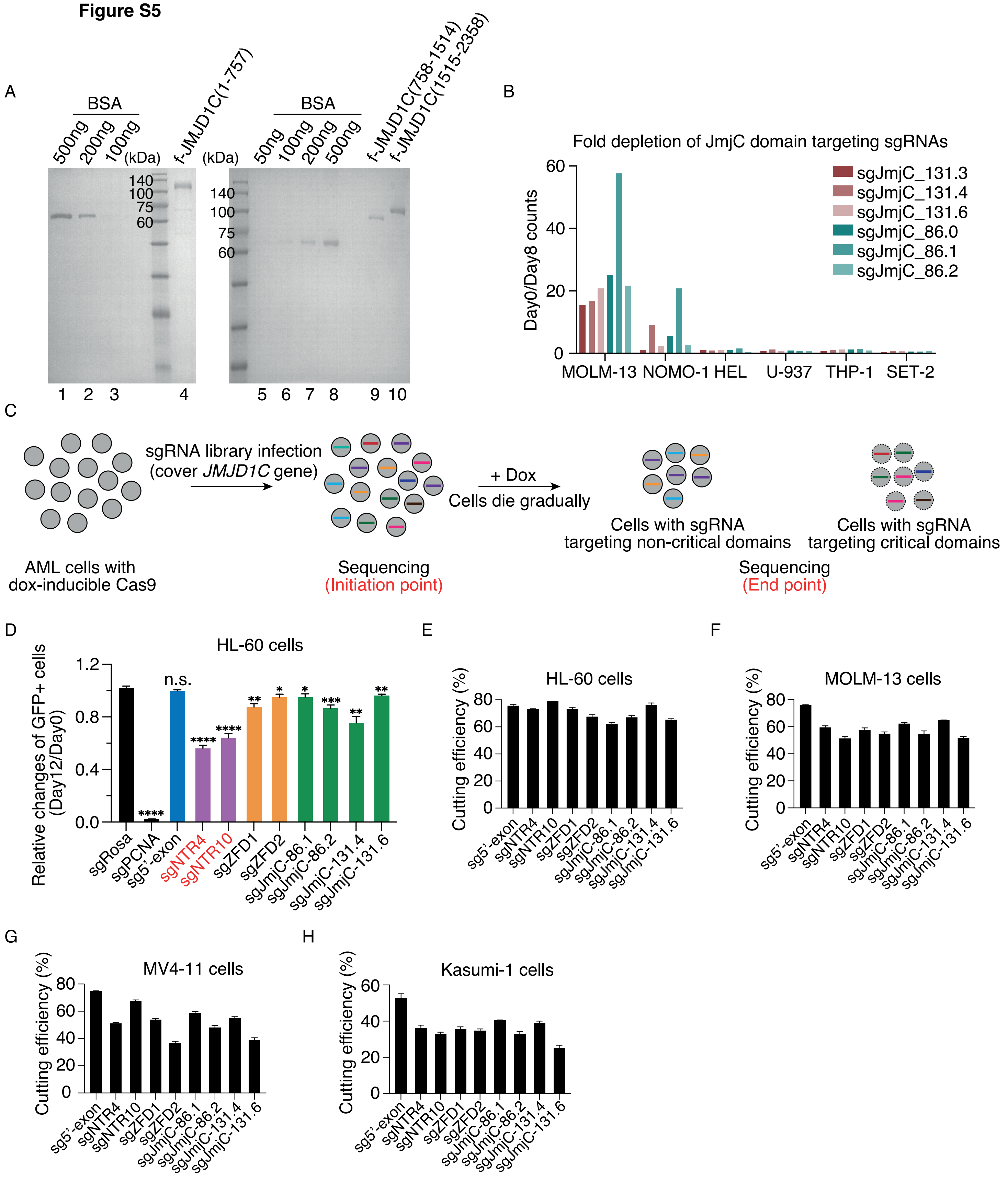
A
BSA
500ng
200ng
100ng
(kDa)
f-JMJD1C(1-757)
140
100
75
60
1 2 3 4
(kDa)
BSA
50ng
100ng
200ng
500ng
f-JMJD1C(758-1514)
f-JMJD1C(1515-2358)
140
100
75
60
5 6 7 8 9 10
B
Fold depletion of JmjC domain targeting sgRNAs
Day0/Day8 counts
sgJmjC_131.3
sgJmjC_131.4
sgJmjC_131.6
sgJmjC_86.0
sgJmjC_86.1
sgJmjC_86.2
MOLM-13 NOMO-1 HEL U-937 THP-1 SET-2
C
sgRNA library infection
(cover JMJD1C gene)
+ Dox
Cells die gradually
AML cells with
dox-inducible Cas9
Sequencing
(Initiation point)
Cells with sgRNA
targeting non-critical domains
Cells with sgRNA
targeting critical domains
Sequencing
(End point)
D
HL-60 cells
Relative changes of GFP+ cells
(Day12/Day0)
n.s.
sgRosa
sgPCNA
sg5'-exon
sgNTR4
sgNTR10
sgZFD1
sgZFD2
sgJmjC-86.1
sgJmjC-86.2
sgJmjC-131.4
sgJmjC-131.6
E
HL-60 cells
Cutting efficiency (%)
F
MOLM-13 cells
Cutting efficiency (%)
G
MV4-11 cells
Cutting efficiency (%)
H
Kasumi-1 cells
Cutting efficiency (%)

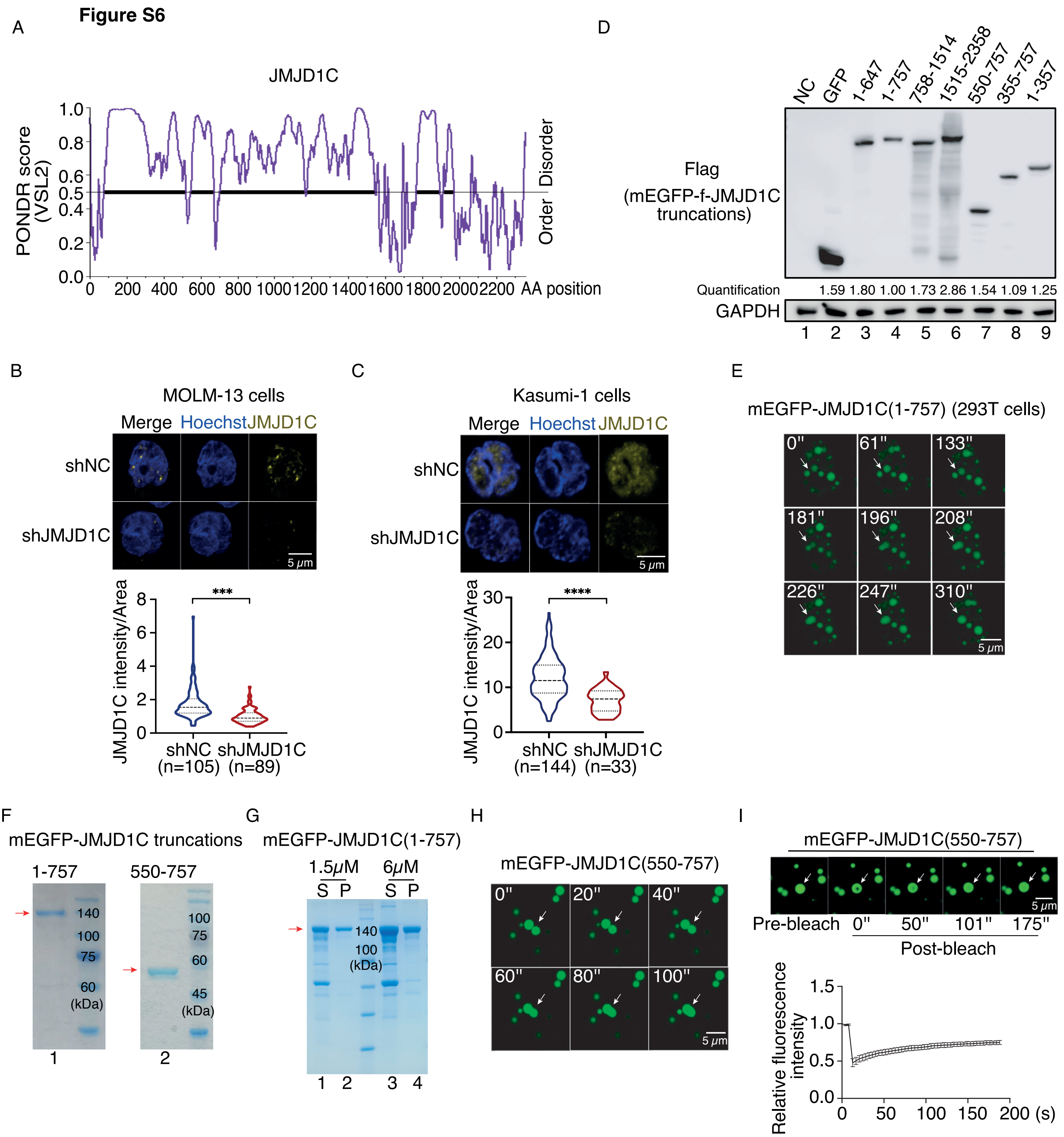
Figure S6
A
JMJD1C
PONDR score (VSL2)
Disorder
Order
AA position
D
NC GFP 1-647 1-757 758-1514 1515-2358 550-757 355-757 1-357
Flag (mEGFP-f-JMJD1C truncations)
Quantification 1.59 1.80 1.00 1.73 2.86 1.54 1.09 1.25
GAPDH
1 2 3 4 5 6 7 8 9
B
MOLM-13 cells
Merge Hoechst JMJD1C
shNC
shJMJD1C
5 μm
JMJD1C intensity/Area
***
shNC (n=105)
shJMJD1C (n=89)
C
Kasumi-1 cells
Merge Hoechst JMJD1C
shNC
shJMJD1C
5 μm
JMJD1C intensity/Area
****
shNC (n=144)
shJMJD1C (n=33)
E
mEGFP-JMJD1C(1-757) (293T cells)
0'' 61'' 133'' 181'' 196'' 208'' 226'' 247'' 310''
5 μm
F
mEGFP-JMJD1C truncations
1-757
550-757
140 100 75 60 (kDa)
100 75 60 45 (kDa)
1
2
G
mEGFP-JMJD1C(1-757)
1.5μM 6μM
S P S P
140 100 (kDa)
1 2 3 4
H
mEGFP-JMJD1C(550-757)
0'' 20'' 40'' 60'' 80'' 100''
5 μm
I
mEGFP-JMJD1C(550-757)
Pre-bleach 0'' 50'' 101'' 175''
Post-bleach
5 μm
Relative fluorescence intensity
(s)

Figure S7

A

	LLPS ability	RUNX1 interaction
1-757	Yes	Yes
550-757	Weak	Very weak
355-757	Weak	Very weak
138-757	No	No
120-757	No	Weak
100-757	No	Weak
80-757	No	Weak
60-757	No	Weak
40-757	No	Weak
30-757	No	Weak
15-757	Yes	Yes
1-137	No	Very weak
1-357	No	Weak
1-647	No	Yes

B

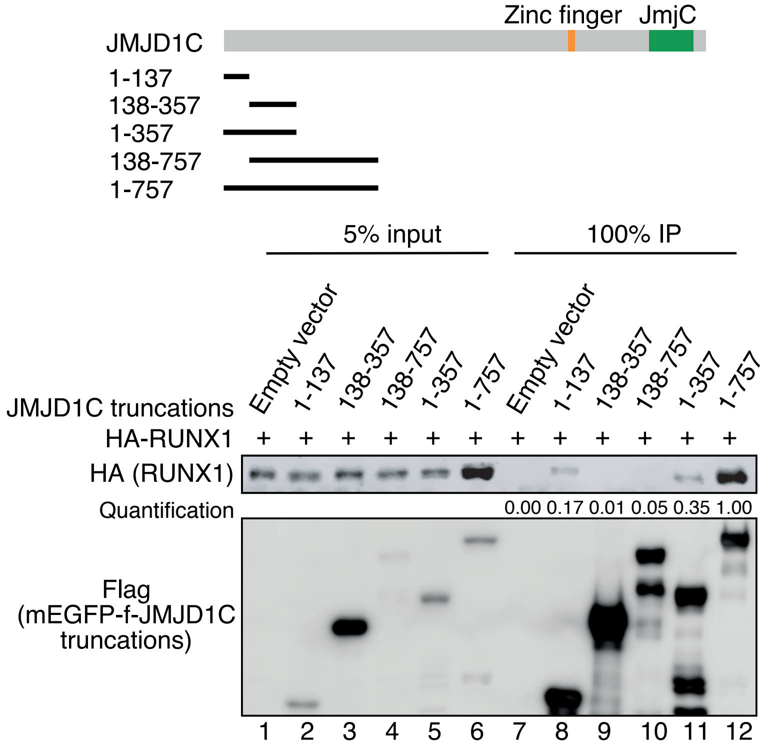

C

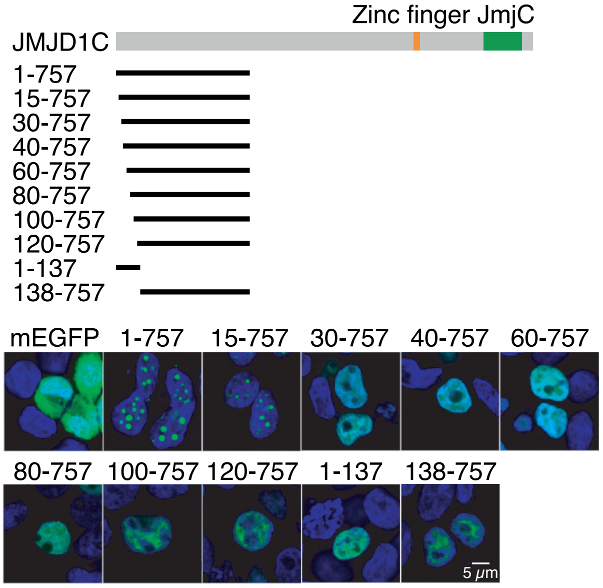

D

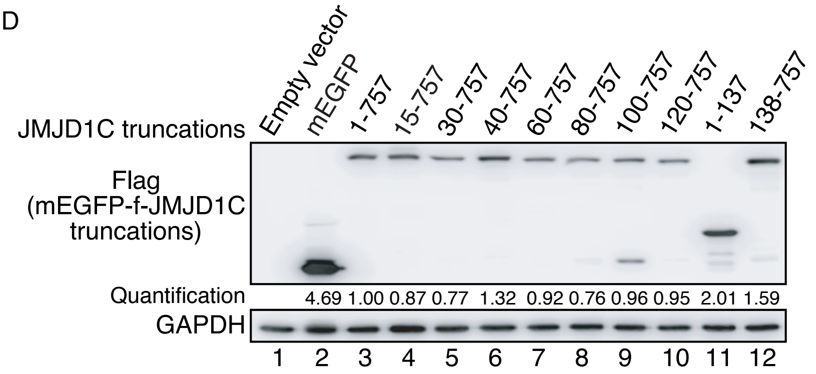

E

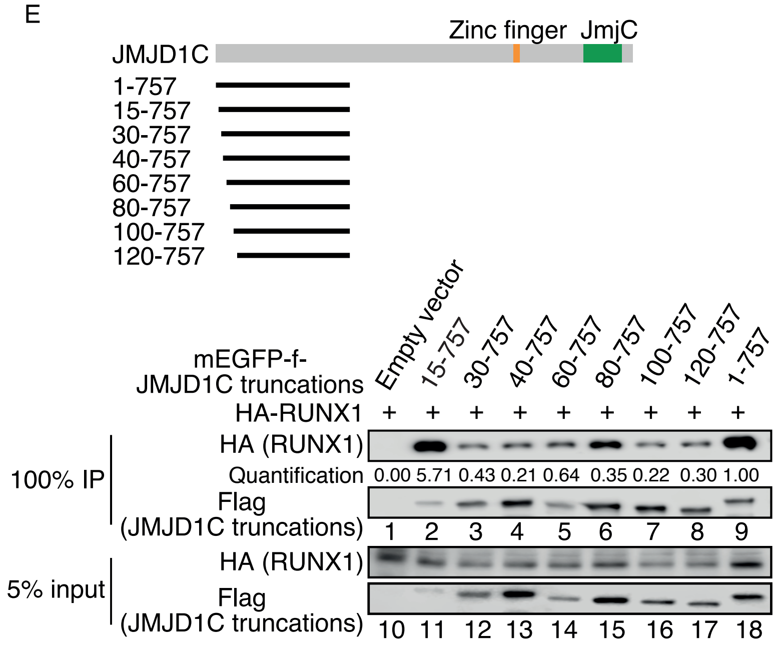

F

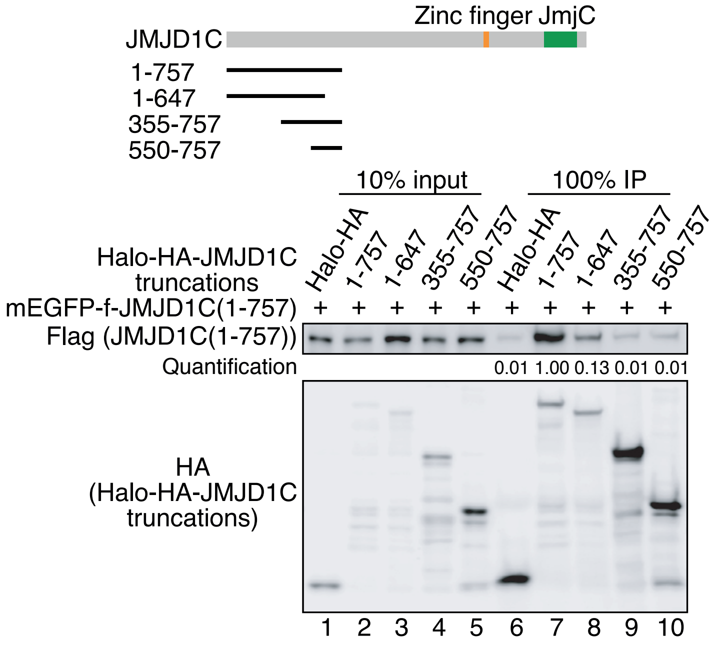

Figure S8

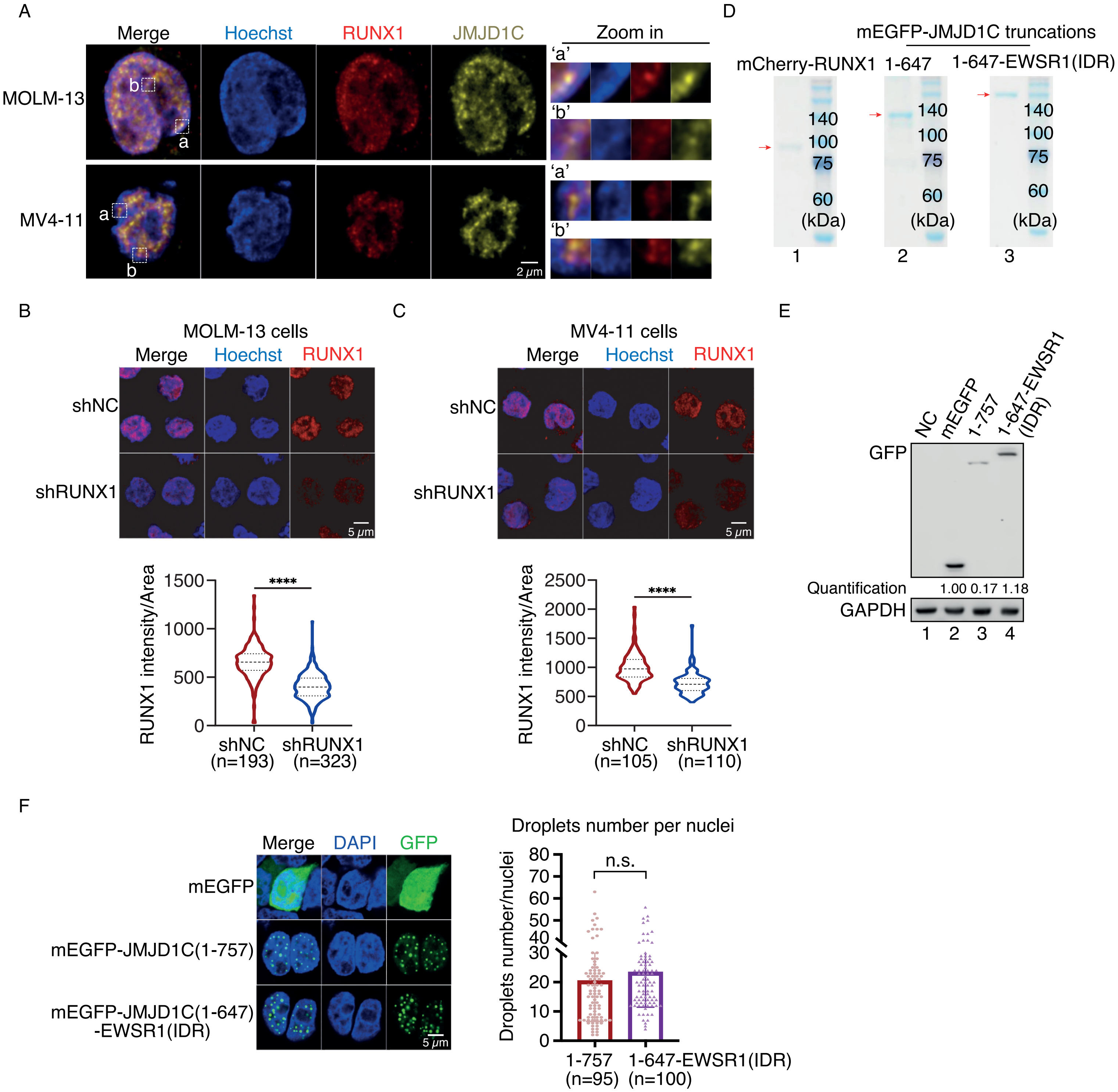
A
Merge
Hoechst
RUNX1
JMJD1C
Zoom in
'a'
'b'
MOLM-13
MV4-11
a
b
2 μm
D
mEGFP-JMJD1C truncations
mCherry-RUNX1
1-647
1-647-EWSR1(IDR)
140
100
75
60
(kDa)
1
2
3
B
MOLM-13 cells
Merge
Hoechst
RUNX1
shNC
shRUNX1
5 μm
RUNX1 intensity/Area
1500
1000
500
0
****
shNC (n=193)
shRUNX1 (n=323)
C
MV4-11 cells
Merge
Hoechst
RUNX1
shNC
shRUNX1
5 μm
RUNX1 intensity/Area
2500
2000
1500
1000
500
0
****
shNC (n=105)
shRUNX1 (n=110)
E
NC
mEGFP
1-757
1-647-EWSR1 (IDR)
GFP
Quantification
1.00 0.17 1.18
GAPDH
1 2 3 4
F
Merge
DAPI
GFP
mEGFP
mEGFP-JMJD1C(1-757)
mEGFP-JMJD1C(1-647) -EWSR1(IDR)
5 μm
Droplets number per nuclei
Droplets number/nuclei
80
70
60
50
40
30
20
10
0
n.s.
1-757 (n=95)
1-647-EWSR1(IDR) (n=100)

Figure S9

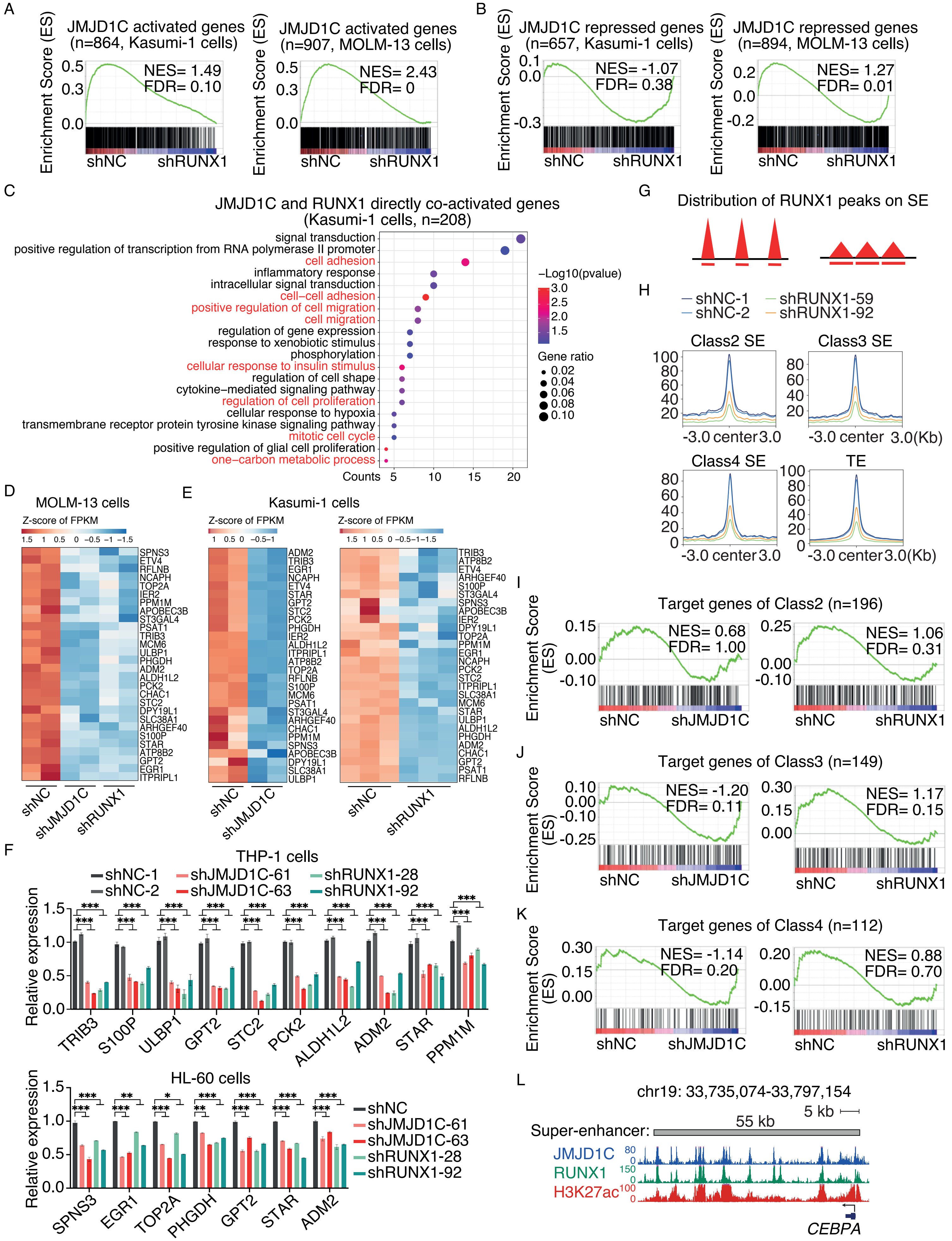
A
JMJD1C activated genes
(n=864, Kasumi-1 cells)
NES= 1.49
FDR= 0.10
JMJD1C activated genes
(n=907, MOLM-13 cells)
NES= 2.43
FDR= 0
Enrichment Score (ES)
shNC
shRUNX1
B
JMJD1C repressed genes
(n=657, Kasumi-1 cells)
NES= -1.07
FDR= 0.38
JMJD1C repressed genes
(n=894, MOLM-13 cells)
NES= 1.27
FDR= 0.01
C
JMJD1C and RUNX1 directly co-activated genes
(Kasumi-1 cells, n=208)
signal transduction
positive regulation of transcription from RNA polymerase II promoter
cell adhesion
inflammatory response
intracellular signal transduction
cell−cell adhesion
positive regulation of cell migration
cell migration
regulation of gene expression
response to xenobiotic stimulus
phosphorylation
cellular response to insulin stimulus
regulation of cell shape
cytokine−mediated signaling pathway
regulation of cell proliferation
cellular response to hypoxia
transmembrane receptor protein tyrosine kinase signaling pathway
mitotic cell cycle
positive regulation of glial cell proliferation
one−carbon metabolic process
Counts
−Log10(pvalue)
Gene ratio
D
MOLM-13 cells
Z-score of FPKM
SPNS3 ETV4 RFLNB NCAPH TOP2A IER2 PPM1M APOBEC3B ST3GAL4 PSAT1 TRIB3 MCM6 ULBP1 PHGDH ADM2 ALDH1L2 PCK2 CHAC1 STC2 DPY19L1 SLC38A1 ARHGEF40 S100P STAR ATP8B2 GPT2 EGR1 ITPRIPL1
shNC
shJMJD1C
shRUNX1
E
Kasumi-1 cells
Z-score of FPKM
ADM2 TRIB3 EGR1 NCAPH ETV4 STAR GPT2 STC2 PCK2 PHGDH IER2 ALDH1L2 ITPRIPL1 ATP8B2 TOP2A RFLNB S100P MCM6 PSAT1 ST3GAL4 ARHGEF40 CHAC1 PPM1M SPNS3 APOBEC3B DPY19L1 SLC38A1 ULBP1
TRIB3 ATP8B2 ETV4 ARHGEF40 S100P ST3GAL4 SPNS3 APOBEC3B IER2 DPY19L1 TOP2A PPM1M EGR1 NCAPH PCK2 STC2 ITPRIPL1 SLC38A1 MCM6 STAR ULBP1 ALDH1L2 PHGDH ADM2 CHAC1 GPT2 PSAT1 RFLNB
shNC
shJMJD1C
shRUNX1
F
THP-1 cells
shNC-1
shNC-2
shJMJD1C-61
shJMJD1C-63
shRUNX1-28
shRUNX1-92
Relative expression
TRIB3 S100P ULBP1 GPT2 STC2 PCK2 ALDH1L2 ADM2 STAR PPM1M
HL-60 cells
shNC
shJMJD1C-61
shJMJD1C-63
shRUNX1-28
shRUNX1-92
SPNS3 EGR1 TOP2A PHGDH GPT2 STAR ADM2
G
Distribution of RUNX1 peaks on SE
H
shNC-1
shNC-2
shRUNX1-59
shRUNX1-92
Class2 SE
Class3 SE
Class4 SE
TE
-3.0 center 3.0 (Kb)
I
Target genes of Class2 (n=196)
NES= 0.68
FDR= 1.00
NES= 1.06
FDR= 0.31
shNC
shJMJD1C
shRUNX1
J
Target genes of Class3 (n=149)
NES= -1.20
FDR= 0.11
NES= 1.17
FDR= 0.15
K
Target genes of Class4 (n=112)
NES= -1.14
FDR= 0.20
NES= 0.88
FDR= 0.70
L
chr19: 33,735,074-33,797,154
5 kb
55 kb
Super-enhancer:
JMJD1C
RUNX1
H3K27ac
CEBPA

Figure S10

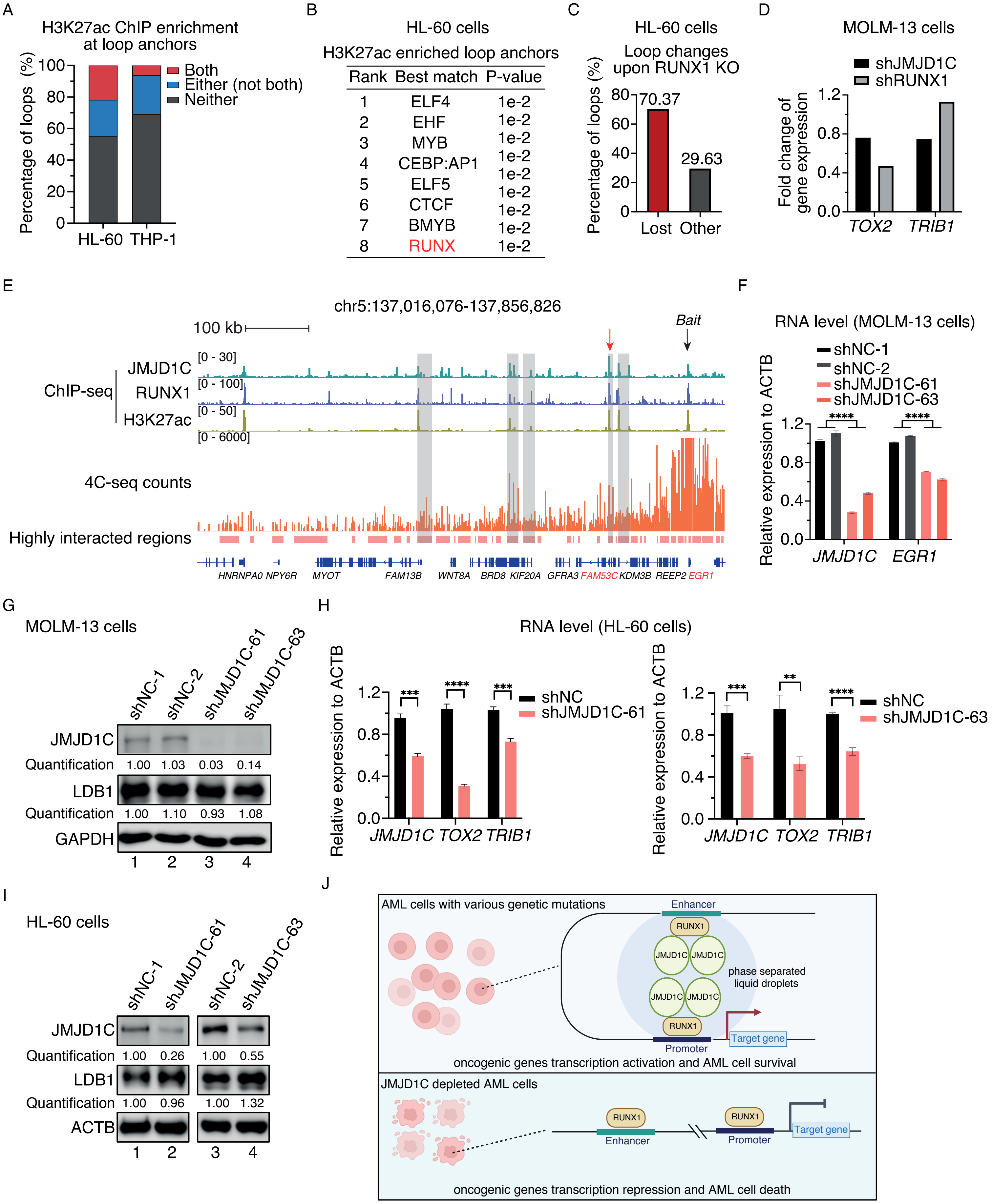
A
H3K27ac ChIP enrichment at loop anchors
Percentage of loops (%)
Both
Either (not both)
Neither
HL-60
THP-1
B
HL-60 cells
H3K27ac enriched loop anchors
Rank Best match P-value
1 ELF4 1e-2
2 EHF 1e-2
3 MYB 1e-2
4 CEBP:AP1 1e-2
5 ELF5 1e-2
6 CTCF 1e-2
7 BMYB 1e-2
8 RUNX 1e-2
C
HL-60 cells
Loop changes upon RUNX1 KO
Percentage of loops (%)
70.37
29.63
Lost
Other
D
MOLM-13 cells
shJMJD1C
shRUNX1
Fold change of gene expression
TOX2
TRIB1
E
chr5:137,016,076-137,856,826
100 kb
Bait
ChIP-seq
JMJD1C
RUNX1
H3K27ac
[0 - 30]
[0 - 100]
[0 - 50]
[0 - 6000]
4C-seq counts
Highly interacted regions
HNRNPA0 NPY6R MYOT FAM13B WNT8A BRD8 KIF20A GFRA3 FAM53C KDM3B REEP2 EGR1
F
RNA level (MOLM-13 cells)
shNC-1
shNC-2
shJMJD1C-61
shJMJD1C-63
Relative expression to ACTB
JMJD1C
EGR1
G
MOLM-13 cells
shNC-1
shNC-2
shJMJD1C-61
shJMJD1C-63
JMJD1C
Quantification 1.00 1.03 0.03 0.14
LDB1
Quantification 1.00 1.10 0.93 1.08
GAPDH
1 2 3 4
H
RNA level (HL-60 cells)
Relative expression to ACTB
shNC
shJMJD1C-61
shJMJD1C-63
JMJD1C TOX2 TRIB1
I
HL-60 cells
shNC-1
shJMJD1C-61
shNC-2
shJMJD1C-63
JMJD1C
Quantification 1.00 0.26 1.00 0.55
LDB1
Quantification 1.00 0.96 1.00 1.32
ACTB
1 2 3 4
J
AML cells with various genetic mutations
Enhancer
RUNX1
JMJD1C
phase separated liquid droplets
Promoter
Target gene
oncogenic genes transcription activation and AML cell survival
JMJD1C depleted AML cells
oncogenic genes transcription repression and AML cell death
